# Mantis Leg-Inspired Smart Insole Integrating Closed-Loop Power Supply for Advanced Wearable Gait Diagnostics

**DOI:** 10.34133/research.1063

**Published:** 2026-01-08

**Authors:** Yingchun Li, Yarong Ding, Yuze Zhang, Xing Guo, Kaixin Lei, Jiachun Sun, Xing Hu, Xinyue Li, Wenguang Yang, Rui Liu, Zhenhua Lin, Wendong Zhang, Shaozhe Tan, Xu Yang, Yumeng Xu, Jin Tian, Bokun Zhang, Yue Hao, Xiangning Li, Yannan Liu, Feng Xu, Jingjing Chang

**Affiliations:** ^1^ Advanced Interdisciplinary Research Center for Flexible Electronics, Academy of Advanced Interdisciplinary Research, Xidian University, Xi’an 710071, P.R. China.; ^2^ State Key Laboratory of Wide-Bandgap Semiconductor Devices and Integrated Technology, Faculty of Integrated Circuit, Xidian University, Xi’an 710071, P.R. China.; ^3^ Institute of Electromechanical Science and Technology, Xidian University, Xi’an 710071, P.R. China.; ^4^ Department of Rehabilitation Medicine, Tangdu Hospital, Air Force Military Medical University, Xi’an 710032, P.R. China.; ^5^ Shaanxi Key Laboratory of Degradable Biomedical Materials, School of Chemical Engineering, Northwest University, Xi’an 710069, P.R. China.; ^6^ The Key Laboratory of Biomedical Information Engineering of Ministry of Education, School of Life Science and Technology, Xi’an Jiaotong University, Xi’an 710049, P.R. China.; ^7^ Bioinspired Engineering and Biomechanics Center (BEBC), Xi’an Jiaotong University, Xi’an 710049, P.R. China.; ^8^ School of Sport and Physical Education, Jiaotong University, Xi’an 710049, P.R. China.

## Abstract

Precise diagnosis and management of lower extremity dysfunction disorders hinge on continuous gait monitoring. Nevertheless, the existing wearable devices fall short as they grapple with insufficient sensing precision, inadequate energy endurance, and ineffective intelligent data analysis. Here, we report a fully integrated, biomimetic smart insole that incorporates 3 synergistic innovations to overcome these challenges. First, inspired by the hierarchical mechanosensory apparatus of mantis legs, we design dual-microstructure capacitive sensors with a detection limit of 0.10 Pa and a maximum detection range of 1.4 MPa. This sensor can distinguish pressures across a wide range from subtle to substantial and exhibits robust mechanical stability over 12,000 cycles, making it highly suitable for insole applications and outperforming current flexible pressure sensors. Second, we realize energy-autonomous operation by integrating nano-perovskite solar cells with high-capacity lithium–sulfur nanobatteries, achieving an average photocharging efficiency of 11.21% and energy storage efficiency of 72.15%. Third, embedded artificial intelligence algorithms interpret the spatiotemporal pressure data transmitted via a 16-channel wireless module. These models achieve 96.0% accuracy in detecting foot arch abnormalities and 97.6% accuracy in classifying 12 pathological gait patterns. Collectively, these 3 advances, including bioinspired high-resolution sensing, sustainable energy interfacing, and intelligent mechanodiagnosis, establish a closed-loop wearable platform validated in clinical studies. This system offers promising applications in early disease screening, personalized rehabilitation, and remote healthcare.

## Introduction

Gait disorders are becoming increasingly prevalent due to population aging, chronic diseases, and foot deformities. This growing incidence highlights an urgent clinical need for efficient gait monitoring [1,2]. Gait analysis is widely recognized as a sensitive and quantitative biomarker for lower extremity functional status, providing actionable insights into both disease trajectory and rehabilitation outcomes [3,4]. However, conventional clinical gait assessment remains confined to controlled laboratory settings due to the dependence on optical motion-capture systems and force plate technologies that are capital-intensive, spatially restrictive, and inherently incapable of capturing naturalistic movement patterns [5,6]. Wearable pressure-sensing insoles have the potential to decentralize and democratize gait diagnostics, yet existing solutions struggle to concurrently fulfill 3 interdependent criteria for clinical translation: (a) sensing elements with the durability and resolution to span the entire biomechanical load spectrum, (b) energy autonomy to sustain uninterrupted data capture, and (c) on-device intelligence for real-time extraction of pathological gait signatures [7,8].

Current flexible pressure sensors are constrained by intrinsic performance trade-offs. The plantar interface imposes extreme and heterogeneous mechanical loads: transient impact forces exceeding 1 MPa during athletic maneuvers, minute quasi-static variations (<1 Pa) during postural control, and high-frequency cyclic loading across tens of thousands of daily steps, a demand regime that rapidly degrades conventional sensors with homogeneous or single-scale architectures [9,10]. Among various transduction mechanisms, capacitive sensing was selected owing to its superior signal stability, low power consumption, and resistance to temperature or humidity interference, making it highly suitable for continuous gait monitoring compared with resistive, piezoresistive, or piezoelectric counterparts [11]. Although bioinspired paradigms, such as gecko-inspired hierarchical stress distribution or spider-slit-sensilla-mimetic vibration transduction, have enhanced dynamic responsiveness [12–14], these strategies often trade mechanical robustness (>10,000 cycles) or long-term biocompatibility for performance gains, limiting their suitability for chronic use.

Energy autonomy constitutes an equally formidable bottleneck. High-density sensor arrays and continuous wireless data streaming impose substantial and sustained power demands, whereas bulky batteries or frequent recharging interrupt the continuity required to capture episodic and transient gait anomalies [15,16]. While emerging modalities such as biofuel cells and triboelectric nanogenerators (TENGs) offer promising alternatives to conventional batteries, biofuel cells are prone to enzymatic degradation and biofouling in perspiration, and TENGs, although capable of high-voltage generation, produce low-current outputs insufficient for modern wireless modules [17–19]. Recent breakthroughs in flexible perovskite solar cells (PSCs), capable of delivering high photovoltaic efficiency under both outdoor sunlight and indoor illumination, open a viable pathway to truly self-sustaining wearable systems [20–22]. When strategically coupled with high-energy-density lithium–sulfur (Li–S) batteries, PSCs can form a closed-loop, environmentally adaptive energy infrastructure resilient to variable operating conditions [23]. However, no integrated platform has successfully unified these technologies to enable seamless operation across the full spectrum of real-world environments, from dimly lit interiors to dynamic outdoor settings.

Beyond hardware, the translation of raw biomechanical data into clinically actionable intelligence remains a critical and underaddressed frontier [24]. Although machine learning frameworks can achieve high classification accuracies in controlled trials [25], most existing systems lack embedded, low-latency analytics, intuitive visualization interfaces, and adaptive feedback mechanisms—capabilities that are essential for maximizing patient compliance and supporting real-time clinical decision-making [26]. A transformative gait-monitoring platform must therefore integrate continuous high-fidelity sensing with artificial intelligence (AI) models trained on heterogeneous pathological datasets, delivering autonomous, point-of-care diagnostic capabilities that extend well beyond laboratory constraints [27].

**Fig. 1.**
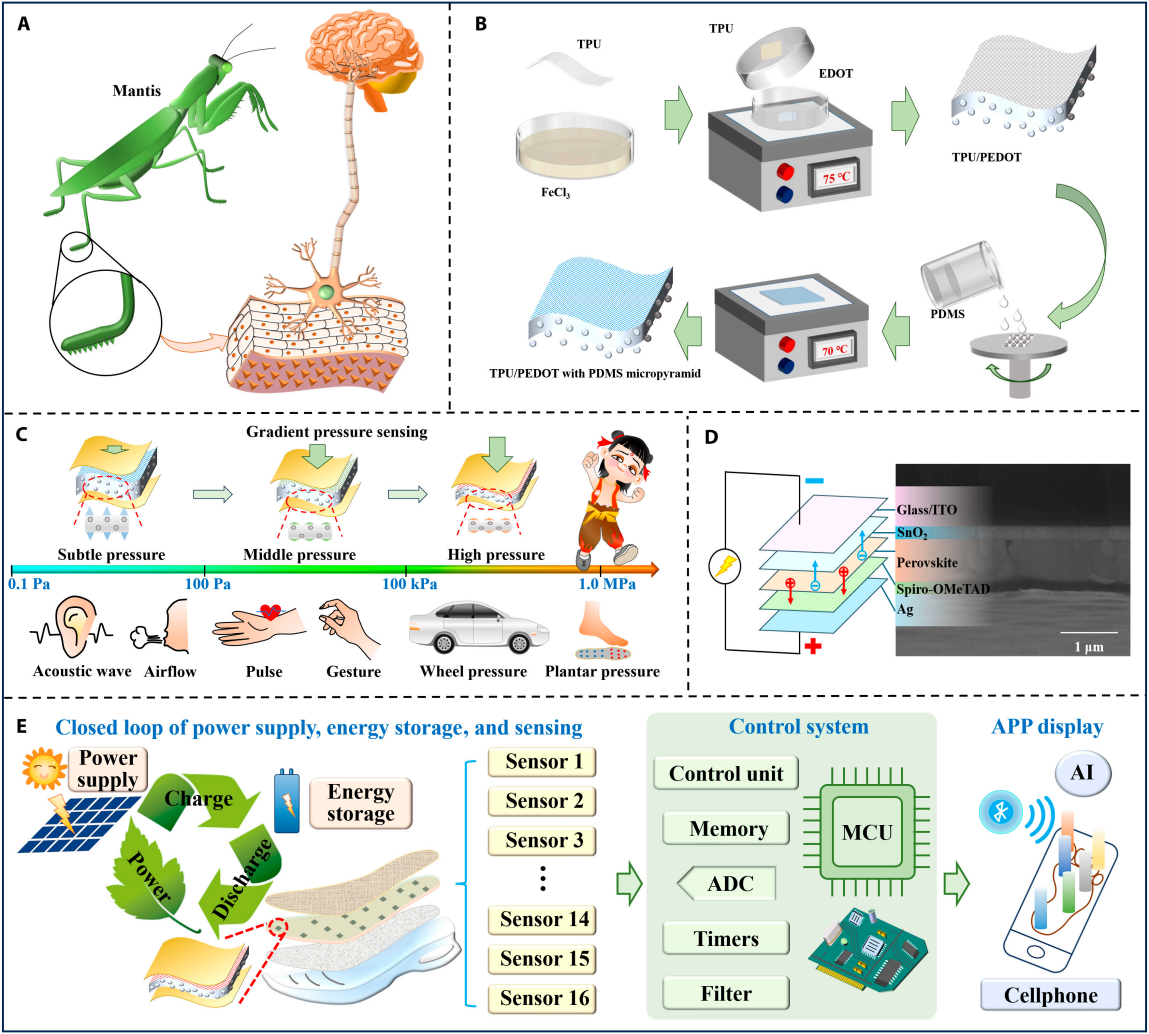
Schematic illustration of energy-autonomous biomimetic insole with AI-enhanced mechanodiagnosis for continuous gait monitoring. (A) Sensing inspiration from mantis legs. (B) Schematic illustration of the fabrication process and features of the dielectric layers. (C) Sensing mechanism of the as-designed capacitive pressure sensor for gradient pressure sensing. (D) Schematic diagram and SEM image of PSC device and perovskite crystal structure. (E) Schematic illustration of the integrated smart insole of sensing–power supply–AI diagnosis for gait monitoring.

Here, we report a biomimetic smart insole that overcomes these convergent challenges through 3 co-engineered innovations (Fig. 1). First, inspired by the hierarchical mechanosensory apparatus of mantis legs (Fig. 1A) [28], we developed a dual-microstructure capacitive pressure sensor that synergistically integrates polydimethylsiloxane (PDMS) microstructures with compressible elastomeric foam (Fig. 1B). This design achieves an ultralow detection limit (0.10 Pa), exceptional sensitivity (0.602 kPa^−1^), and a broad dynamic range (0.10 Pa to 1.40 MPa) while maintaining mechanical endurance beyond 12,000 loading cycles (Fig. 1C). Second, a hybrid PSC/Li–S energy system ensures uninterrupted operation across diverse environments, eliminating dependency on external power infrastructure (Fig. 1D and E). Third, an embedded AI framework synergizes a random forest classifier for foot arch abnormality detection (96% accuracy) and a one-dimensional convolutional neural network (1D-CNN) for classifying 12 pathological gait patterns (97.6% accuracy). A companion mobile APP visualizes dynamic force fields through chromatic mapping, empowering clinicians with real-time, interpretable diagnostics (Fig. 1E). By integrating high-fidelity biomechanical sensing, self-sufficient energy harvesting, and edge-computing intelligence, this platform bridges a critical gap between wearable technology and clinical practice. Its ability to provide continuous, AI-augmented gait analysis outside laboratory settings advances preventive care paradigms, from proactive fall-risk mitigation in geriatric populations to personalized, data-driven rehabilitation in telemedicine ecosystems.

## Results and Discussion

### Fabrication and characterization of dual-microstructure sensors

The trunk morphology and surface spines of mantis legs are closely related to the mechanical properties of the leg material, which in turn enable mantis legs to exhibit strong jumping ability and precise recognition capability during movement and predation [28]. To enable continuous detection of gait-related pressures from subtle postural shifts to high-impact forces, we engineered a capacitive pressure sensor as inspired by the hierarchical elasticity of mantis legs (Fig. 1A). The sensor integrates 2 key components of thermoplastic polyurethane (TPU) foam doped with poly(3,4-ethylenedioxythiophene) (PEDOT) via vapor-phase polymerization (VPP) and PDMS pyramid microstructures on the surfaces (Fig. 1B). In detail, the TPU foams were first immersed in ethanol solution with 5 % (w/v) FeCl_3_ for 5 min and then dried at 70 °C. For VPP, a device was set up where TPU foam was attached to a petri dish lid, and EDOT monomer was dripped onto the filter paper of the same size as the TPU foam at the base to ensure uniform evaporation and dispersion of the vapor over the TPU surface. The device was heated at 75 °C for 2 h, with the foam inverted at the 1-h mark to achieve uniform polymerization. The open-cell structure of TPU facilitated uniform vapor diffusion, ensuring conformal PEDOT coating across both surface and interior pores. Then, PDMS was processed using a photolithography silicon template and cured onto TPU/PEDOT foam to prepare double-sided micropyramid arrays for PEDOT/TPU foams. Pyramid-shaped microstructures were selected due to their high stress concentration and rapid variation in contact area under small pressure, which enhance sensitivity and detection range. The TPU/PEDOT foam and PDMS pyramid respectively mimic the trunk elastic structure and microspines of mantis legs. These key biological traits of mantis legs guided the design of our sensor’s dual microstructure, ensuring that it mimics the advantageous mechanosensory characteristics of the natural prototype (Fig. 1C).

**Fig. 2.**
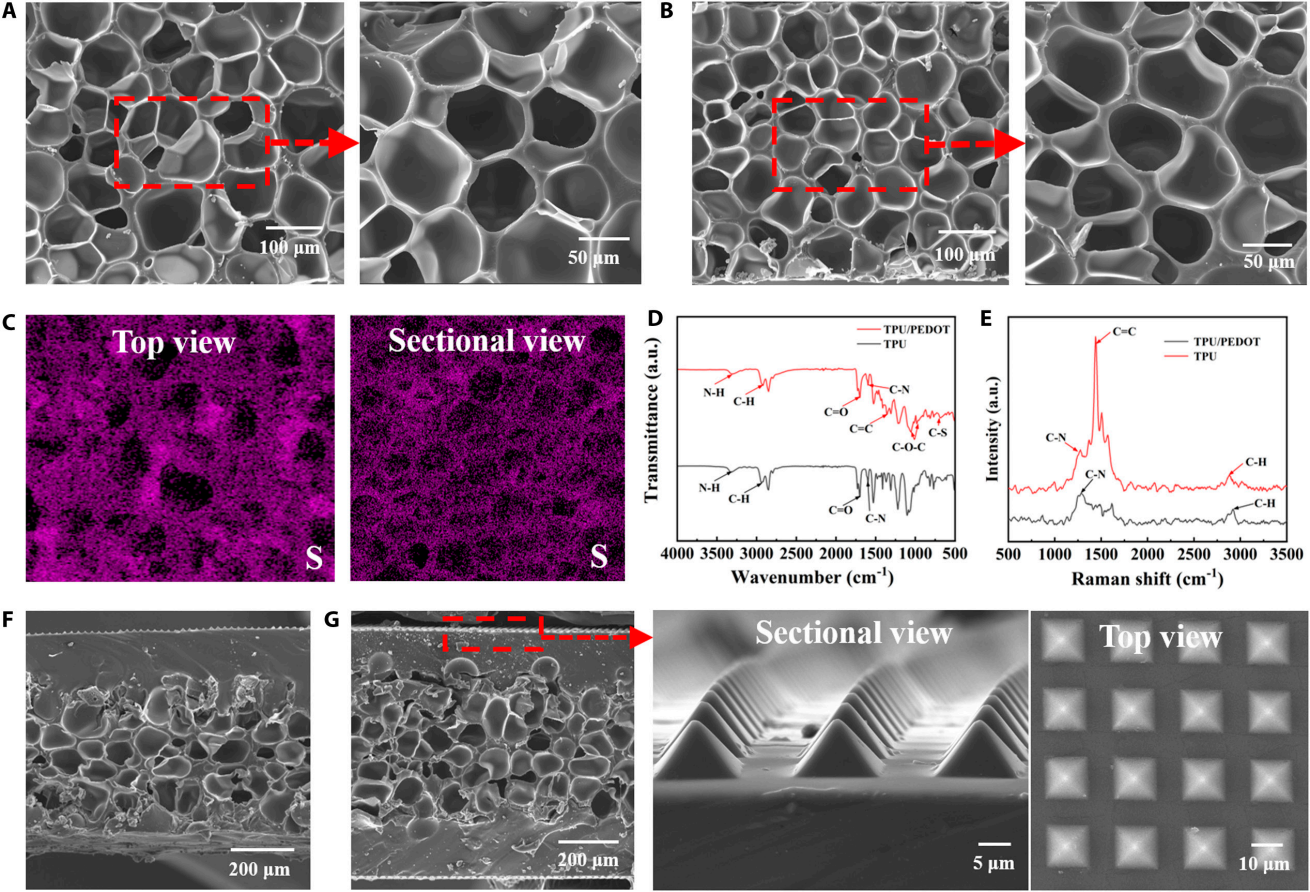
Characterization of the pressure sensor. (A) SEM photograph on the surface and the enlarged view of TPU foam. (B) SEM photograph on the surface and the enlarged view of PEDOT/TPU foam. (C) Mappings of S element on the surface and cross-section of PEDOT/TPU foam. (D) FTIR spectra of the TPU foam and PEDOT/TPU foam. (E) Raman patterns of the TPU foam and PEDOT/TPU foam. (F) SEM photographs on cross-section of PEDOT/TPU foam with single-sided micropyramid array. (G) SEM images and the enlarged view of double-sided micropyramid PEDOT/TPU foam.

To examine the microstructure, we conducted scanning electron microscopy (SEM) imaging of the TPU foam and PEDOT/TPU foam. We observed that both TPU foam and PEDOT/TPU foam exhibit evenly distributed pores with diameters of approximately 40 μm (Fig. 2A and B). Energy-dispersive spectroscopy (EDS) in Fig. S1 and the mappings in Fig. S2 show the intensity and distribution of carbon (C), nitrogen (N), oxygen (O), and sulfur (S) elements across the foam’s surface. Since sulfur is a characteristic element of PEDOT, the uniform distribution of sulfur can be clearly observed in the mappings of the surface and cross-section of TPU/PEDOT in Fig. 2C, which confirms the uniform incorporation of PEDOT into the TPU foam. Fourier transform infrared spectroscopy (FTIR) and Raman spectroscopy further validated the successful polymerization of PEDOT within the TPU matrix. FTIR spectra revealed characteristic peaks at 1,360 cm^−1^ (C=C stretching in the thiophene ring), 1,058 cm^−1^ (asymmetric C–O–C stretching), 899 cm^−1^ (oxyethylene ring deformation), and 689 cm^−1^ (C–S stretching) in the TPU/PEDOT foam, which were absent in pure TPU foam (Fig. 2D). Raman spectra exhibited distinctive PEDOT peaks at 2,930 cm^−1^ (C–H stretching), 1,279 cm^−1^ (C–N stretching), and 1,442 cm^−1^ (C=C stretching), confirming the presence and uniform distribution of PEDOT within the foam (Fig. 2E). In addition, mechanical testing revealed that doping TPU foam with PEDOT markedly enhanced its mechanical properties. As shown in Fig. S3, the Young’s modulus increased from 0.62 MPa for pure TPU foam to 1.19 MPa for TPU/PEDOT foam, indicating improved mechanical strength. Concurrently, the dielectric constant rose from 3.38 to 5.08 after PEDOT incorporation, highlighting enhanced dielectric properties critical for capacitive sensing applications.

To further optimize stress distribution and dielectric modulation, we patterned PDMS pyramids onto the PEDOT-doped foam. Figure 2F shows the SEM image on cross-section of the foam with a single-sided micropyramid array, and Fig. S4 displays the top view under an optical microscope, further demonstrating the uniform distribution of the pyramid arrays. Figure 2G shows the SEM photographs and the enlarged view of PEDOT/TPU foam with double-sided micropyramid array to study the details. It can be observed that the pyramids on both sides have been successfully fabricated, presented completely, and arranged uniformly, with a width of 14 μm, a height of 9 μm, and a spacing of 7 μm between each pyramid. To assess the mechanical properties of the double-sided pyramid sensor, we measured the stress–strain curves, as shown in Fig. S5. When external stresses of 100, 200, and 300 kPa were applied, the stress–strain curves remained largely consistent in shape and trend, with tensile length correlating well with the applied pressure. This result demonstrates that the sensor possesses good and stable mechanical properties, essential for reliable biomechanical sensing.

Thus, the dual-microstructure configuration synergistically combines the high local responsiveness of PDMS pyramids with the mechanical resilience of PEDOT/TPU foam, achieving a favorable balance among sensitivity, durability, and detection range, an essential feature for long-term wearable operation.

### Finite element analysis and sensing performance of the dual-microstructure sensors

**Fig. 3.**
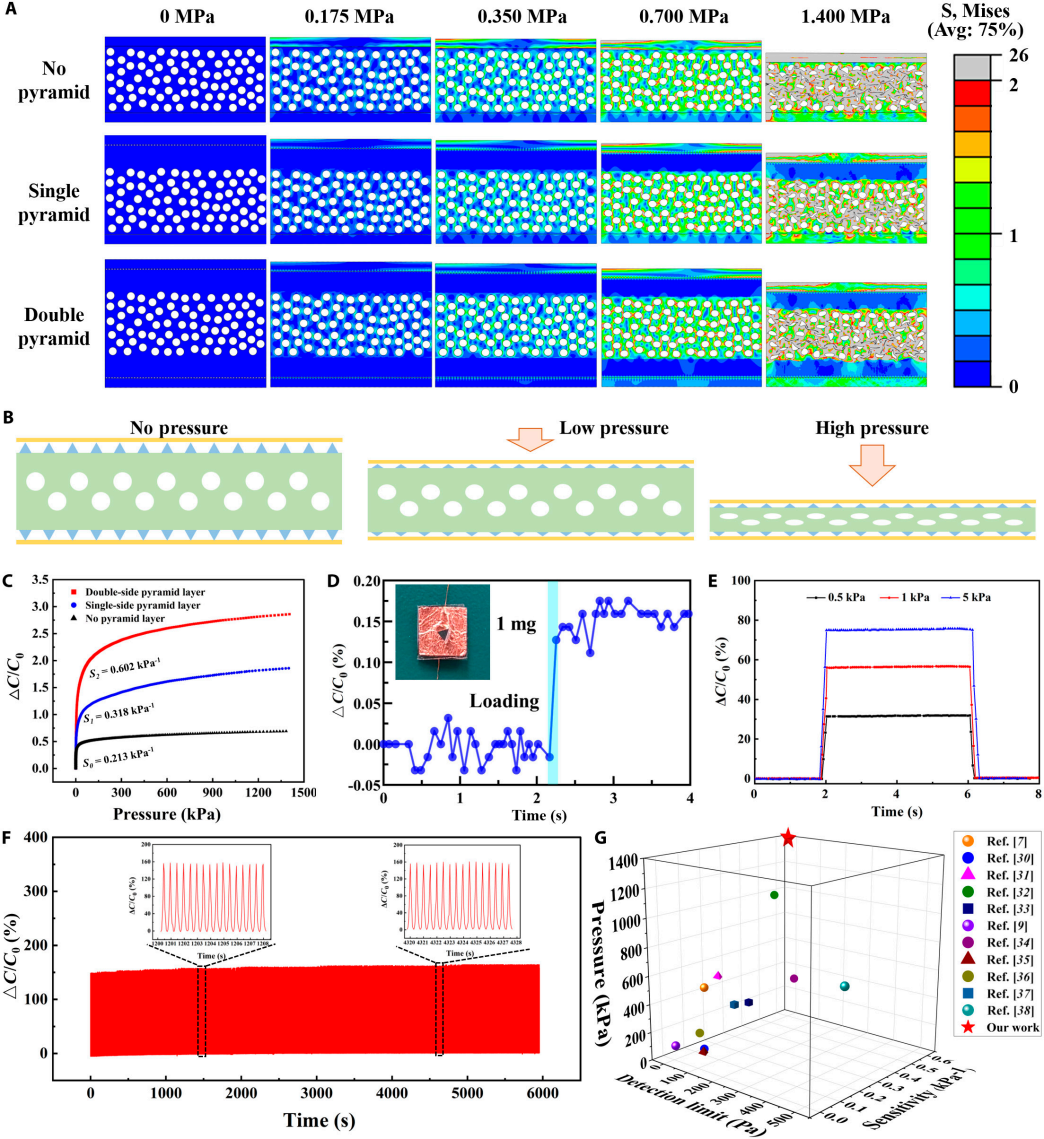
Finite element analysis and performance of the pressure sensor. (A) Finite element simulation of pressing process. (B) Schematic illustration of the pressure sensor with double-sided pyramid microstructures during the pressing process. (C) Sensitivity of sensors without microstructures, with single-sided pyramids, and double-sided pyramids. (D) LOD detection. (E) Response and recovery times at 3 pressure levels. (F) Stability under 100 kPa for 12,000 cycles. (G) Comparison of sensitivity, LOD, and work range among different designs of capacitive pressure sensors.

To simulate the sensing mechanism during compression, we conducted finite element analysis using ABAQUS software (Movie S1). The simulations confirmed that the dual microstructures enhance the air gap effect and dielectric properties, leading to improved sensitivity (Fig. 3A and Fig. S6). Specifically, the pyramid microstructures facilitate greater deformation and contact area changes between the electrodes and the dielectric layer under pressure (Fig. S7A), while the microporous structure of the TPU/PEDOT foam contributes to dielectric constant variation (Fig. S7B).

The schematic diagram in Fig. 3B illustrates the deformation of the pyramid microstructures and PEDOT/TPU foam within the capacitive pressure sensor under external pressure. Under low pressure, the PDMS micro-pyramids and the holes in the dielectric layer were simultaneously compressed. Upon micro-deformation, the PDMS pyramids locally concentrate stress and reduce dielectric spacing, while the PEDOT/TPU foam ensures homogeneous force transfer and electrical continuity, resulting in a synergistically enhanced capacitive response. The deformation enlarges the contact area, reduces electrode spacing, and alters the dielectric constant, thus generating a strong capacitive response.

Experimentally, sensors with double-sided pyramids achieve a sensitivity of 0.602 kPa^−1^, which is 2.8× higher than nonpatterned sensors (0.213 kPa^−1^) and 1.9× higher than single-sided designs (0.318 kPa^−1^) (Fig. 3C). The working range extends to 1.4 MPa, exceeding the peak plantar pressures observed during walking (1.2 MPa) [29]. The experimental results align with simulation findings, consistently demonstrating that the addition of surface microstructures enables the sensor to undergo greater deformation when subjected to force. The tip effect of the pyramid shape leads to stress concentration, resulting in greater deformation under the same force, thereby markedly enhancing the performance. These results demonstrate that our biomimetic microstructures synergistically enhance sensitivity and biomechanical compatibility. To address the clinical need for detecting subtle pressure variations and transient forces, we quantified sensor response times under physiologically relevant loads. The 1-mg object was put on the sensor, estimated to exert approximately 0.1 Pa of pressure, resulting in an increase of △*C*/*C*_0_ of the sensor, demonstrating its ability to accurately identify the pressure of 0.1 Pa, which is the limit of detection (LOD) of the sensor (Fig. 3D). As a result, the TPU/PEDOT foam mimics the compliant, energy-dissipating leg segments, while the PDMS pyramid microstructures replicate the localized stress concentration of mechanosensory hairs, enabling ultralow detection limits and a broad dynamic range.

Furthermore, as shown in Fig. 3E and Fig. S8, the sensor exhibits response/recovery times of 135/127 ms, 167/156 ms, and 192/195 ms under pressures of 0.5, 1, and 5 kPa, respectively. Figure 3F depicts △*C*/*C*_0_ of the sensor during continuous cyclic load under 100-kPa pressure at 2 Hz. The inset shows an enlarged view of △*C*/*C*_0_ of the sensor at different cycle stages. It is evident from the figure that after 12,000 cycles, there is almost no decay in △*C*/*C*_0_, and the amplitude and half-width height at each cycle stage are approximately the same. This demonstrates the sensor’s exceptional stability and robust long-term wearability, making it highly suitable for multi-day gait monitoring. We benchmark our sensor against 10 state-of-the-art flexible pressure sensors [7,9,30–38] across 4 metrics critical for clinical use, including sensitivity (0.602 kPa^−1^), detection limit (0.10 Pa), response time (135 ms), and working range (0.10 Pa to 1.40 MPa) (Fig. 3G and Table S1). The superior performance compared to other flexible sensors can be attributed to the hierarchical deformation of PDMS pyramids and PEDOT/TPU foam, which amplifies capacitance variation while maintaining structural stability under repeated compression, thereby enhancing both sensitivity and durability. The device achieves top-tier performance in combined sensitivity and dynamic range while maintaining mechanical flexibility. This performance profile enables continuous monitoring of pathological gait patterns, such as diabetic foot ulcer precursors and osteoarthritis-related joint overloading.

By combining biomimetic microstructure engineering with conductive polymer doping, we developed a capacitive pressure sensor that overcomes key limitations in wearable gait analysis. The dual-microstructure design achieves clinically relevant sensitivity and durability, while the 0.10-Pa detection limit identifies subclinical biomechanical anomalies.

### Extreme mechanical stress and gradient pressure tolerance of dual-microstructure sensors

**Fig. 4.**
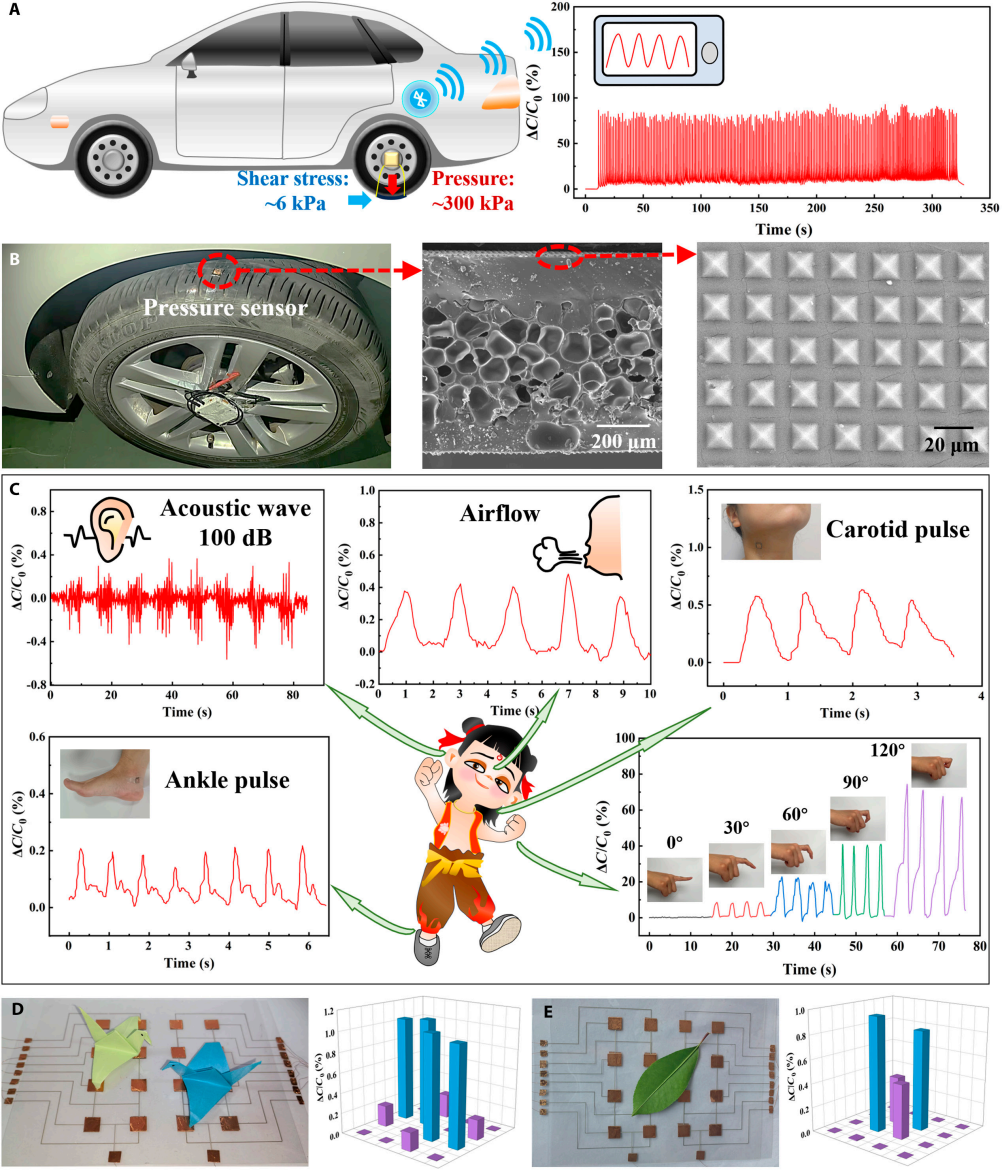
Real-time monitoring application demo of capacitive pressure sensor. (A) Schematic illustration of the testing setup and the signal stability of the pressure sensor affixed to the tire tread during the car running on the road. (B) Photograph of the pressure testing system installed on the automobile tire, as well as the SEM images of the sectional view of the pressure sensor and enlarged view of the micropyramid array after testing. (C) Recognition of acoustic wave, airflow, carotid pulse, ankle pulse, and finger bend using the pressure sensor. (D and E) Recognition of pressure distribution on 4 × 4 sensors array for (D) origami cranes and (E) leaf.

To evaluate sensor durability under real-world mechanical stress, we affixed a 1 cm × 1 cm sensor to a car tire tread, subjecting it to 300-kPa compressive and 6-kPa shear forces during continuous driving (Fig. 4A). We observed stable wireless capacitance signals during prolonged continuous testing, with no signal drift or anomalies (Movie S2). Post-test SEM reveals intact TPU/PEDOT foam pores and undamaged PDMS pyramids (Fig. 4B), confirming that the dual-microstructure design resists delamination and deformation under extreme loads. These results demonstrate the sensor’s robustness for wearable applications requiring prolonged exposure to harsh biomechanical forces, such as athletic monitoring.

Biocompatibility is a critical requirement for wearable biomedical devices to prevent adverse skin reactions and ensure user safety during prolonged use. We conducted comprehensive biocompatibility assessments using L929 fibroblast cells cultured in sensor extraction solutions. Acridine orange/propidium iodide (AO/PI) staining after 1 to 3 d of culture exhibited strong green fluorescence signals, indicative of high cell viability and normal spindle-shaped morphology (Fig. S9). TheCell Counting Kit-8 (CCK-8) assay further confirmed that cell survival rates remained above 90% even at high extraction concentrations of 20 mg ml^−1^ (Fig. S10). To evaluate the sensor’s safety on human skin, we conducted prolonged wear tests by applying the flexible sensor to a volunteer’s arm for 8 h daily over a week (Fig. S11). No marked skin irritation, redness, or allergic reactions were observed, demonstrating the sensor’s biocompatibility and suitability for long-term skin contact. These findings affirm that the materials and construction of our sensors are safe for continuous use in wearable biomedical applications.

To verify the gradient pressure sensing capability, we further conducted the sensor’s response to subtle pressures (e.g., acoustic waves and airflow), moderate pressures (e.g., pulse), and angular-dependent pressures from finger bending (Fig. 4C). The sensor exhibits excellent responsiveness to a 100-dB acoustic wave and airflow at speeds below the human skin’s detection threshold (≈0.50 m s^−1^). The system also accurately resolves carotid pulse harmonics (0.50 to 20 Hz) and discriminates ankle pulse amplitude variations [39]. During gesture recognition, capacitance shifts enable precise differentiation of finger flexion angles (30° to 120°). These results demonstrate the sensor’s ability to detect subclinical biomechanical anomalies, such as early-stage neuropathy or joint stiffness.

To validate spatial resolution for medical diagnostics, we fabricated a 4 × 4 array and map pressure distributions from lightweight objects (1 to 10 Pa). The array accurately identified the spatial pressure profiles of origami cranes and leaf positioned at distinct locations (Fig. 4D and E). This spatial recognition capability highlights the platform’s potential for plantar pressure mapping, localized gait abnormality detection, and targeted orthotic design.

By unifying mechanical resilience, gradient pressure sensing capability, and biocompatibility, our dual-microstructure sensors overcome key limitations in current wearable biomechanical monitors. The 300-kPa durability ensures reliable operation under strenuous activities, while the 0.10-Pa sensitivity enables early detection of plantar pressure anomalies (e.g., uneven load distribution associated with foot deformities or gait disorders), a critical advance for preventive healthcare.

### Continuous operation of wearable biomedical devices via integrated photo-rechargeable system

**Fig. 5.**
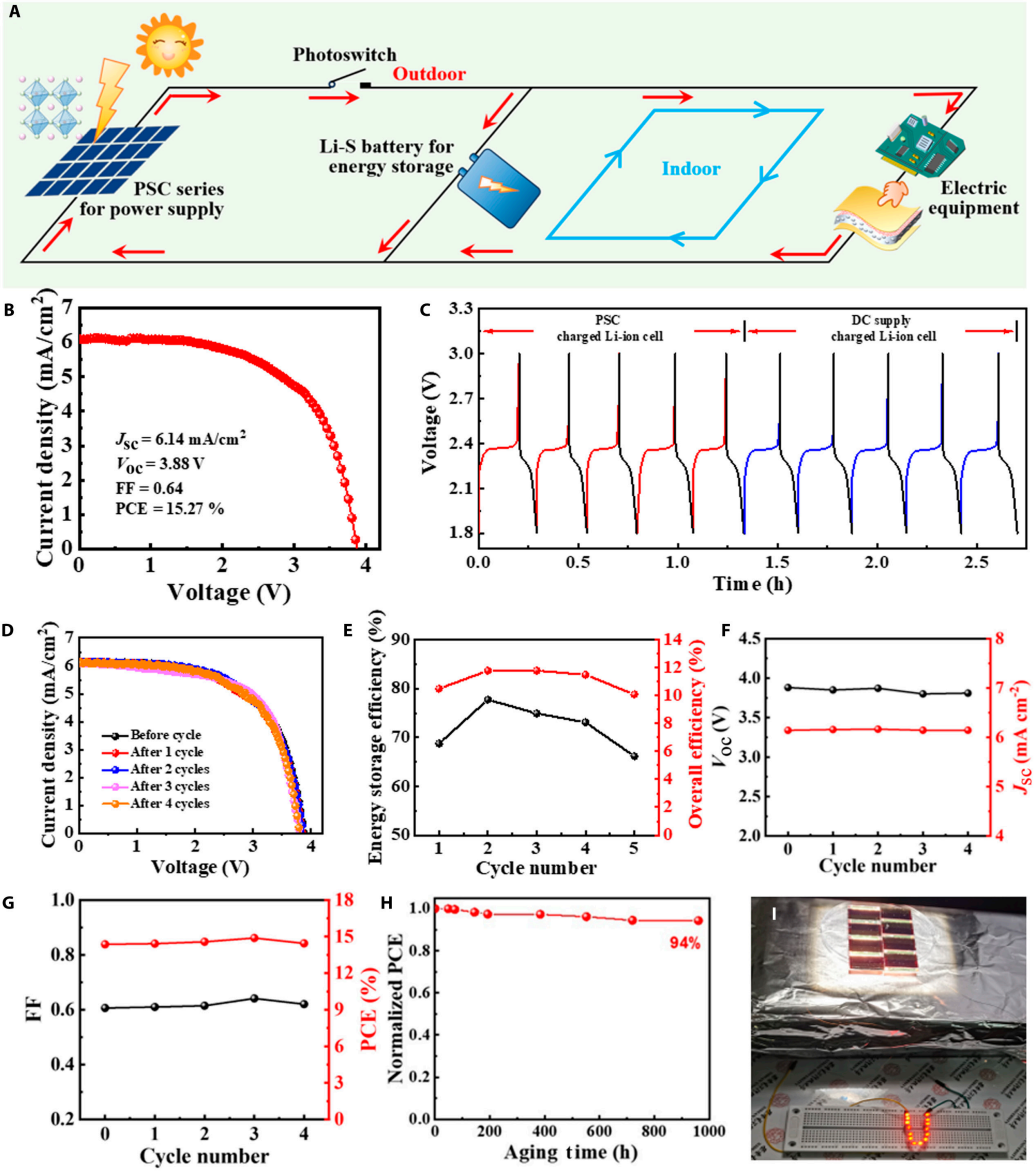
Characterizations of photorechargeable power system. (A) Working principle of closed-loop power supply using PSCs for generation and Li–S batteries for storage. (B) *J–V* curves of 4 PSCs connected in series. (C) Li–S battery voltage profiles during charging (PSC or DC) and discharging. (D) *J–V* curves of 4 series-connected PSCs over 5 cycles. (E) Energy storage and overall efficiency of the PSC-charged Li–S battery. (F and G) Corresponding electrical parameters of (F) *V*_OC_, *J*_SC_, (G) FF, and PCE based on the *J–V* curves of 4 single PSCs connected in series for 5 charge–discharge cycles. (H) Normalized PCE of PSCs as a function of time. (I) LEDs powered by PSC series.

We developed a closed-loop energy system synergizing PSCs with Li–S batteries, where an adaptive energy allocation strategy dynamically switches operation modes across outdoor/indoor environments to sustain uninterrupted wearable monitoring (Fig. 5A). Under high-illumination outdoor conditions, excess photovoltaic output directly charges the Li–S battery, whereas under low-illumination indoor scenarios, the system switches to battery-dominant discharge to sustain sensor and wireless operation. To assess the energy transfer for wearable applications, we connected 4 PSCs in series (3.88 V output) to Li–S batteries with sulfur-infused carbon electrodes. As shown in Fig. 5B, the 4 series-connected PSCs exhibit a power conversion efficiency (PCE) of 15.27% under standard solar illumination (AM1.5G, 100 mW cm^−2^), with a *J*_SC_ of 6.14 mA cm^−2^, a *V*_OC_ of 3.88 V, and a fill factor (FF) of 0.64. Compared to single PSC, the series connection provides the necessary voltage for effective Li–S battery charging. To construct a closed-loop power supply system, we also fabricated the Li–S battery for energy storage with a laminated construction of lithium, electrolyte, separator, and hollow carbon spheres embracing sulfur (Fig. S12).

Galvanostatic charge–discharge cycles reveal nearly identical voltage profiles between PSC-charged and DC-powered systems (Fig. 5C), confirming efficient solar energy conversion. After repeated photocharge–discharge cycles, the Li–S batteries maintain >95% capacity retention and the PSCs show <5% PCE degradation (Fig. 5D). These results confirm the system’s resilience to fluctuating environmental conditions, ensuring uninterrupted power for sensors, wireless modules, and edge-computing units in smart insoles.

The integration of PSCs with Li–S batteries was selected to combine continuous light harvesting with high-capacity energy storage. Unlike TENGs or biofuel cells, PSCs enable stable energy generation under diverse lighting, while Li–S batteries provide superior energy density and cycling life compared to conventional Li-ion batteries, ensuring long-term reliability of the self-powered system. The entire self-charging system is evaluated based on its overall photoelectric conversion efficiency (η_overall_) and energy storage efficiency (η_storage_). The overall photoelectric conversion efficiency of the self-charging system can be calculated as follows:

$$\eta_{\text{overall}}=\frac{E_{d}}{\left(P\times S\times t_{1}\right)}\times 100\%$$ (1)

In the formula, *E_d_*, *P*, *S*, and *t*_1_ represent the discharge energy of the Li–S battery (mWh, measured using the blue energy testing system), light power density (100 mW/cm^2^), effective area of the series-connected PSCs, and the light charging time (h), respectively.

The energy storage efficiency of the self-charging system can be calculated as follows:

$$\eta_{\text{storage}}=\frac{\eta_{\text{overall}}}{\mathrm{PCE}}\times 100\%$$ (2)

In the formula, PCE refers to the PCE of the series-connected PSC module before the charging/discharging cycle.

As a result, the self-charging system achieves an average energy storage efficiency of 72.15% and an average overall photocharging conversion efficiency of 11.14% (Fig. 5E). Furthermore, the stability of PSCs is important for the self-charging system. According to the *J–V* curves of the series-connected PSCs after different photocharge/discharge cycles, the corresponding electrical parameters are shown in Fig. 5F and G. The PSCs exhibit good light stability with almost unchanged electrical properties after long-term illumination over multiple cycles. We exposed the unencapsulated PSC to air with a relative humidity of 40% at room temperature. With 94% efficiency retention over 1,000-h aging, the PSCs enable reliable energy autonomy for wearable systems (Fig. 5H). To further enhance environmental stability, the PSC module was encapsulated using 3M Ultra Barrier Solar Film, achieving a key milestone toward clinical-grade health monitoring. To demonstrate practical utility, we derived 14 commercial light-emitting diodes (LEDs) using 8 series-connected PSCs, achieving stable illumination under simulated sunlight (Fig. 5I and Movie S3).

To address the unmet need for continuous, clinically actionable gait analysis, we integrated 16 high-performance capacitive pressure sensors into an energy-autonomous smart insole, combining a sustainable energy module (PSCs/Li–S batteries), wireless data transmission, and a mobile real-time feedback interface for seamless indoor–outdoor operation with uninterrupted data acquisition. To enable real-time diagnostics, we connected the sensor array to a flexible printed circuit board (FPCB) with Bluetooth 5.0. PSCs and Li–S batteries were affixed to the shoe exterior to eliminate external power reliance, with the control circuit and APP additionally solar-powered for testing (Fig. S13). The system wirelessly streams data to a mobile APP, which generates dynamic heatmaps that visually encode pressure variations through color gradients (Fig. S14 and Movie S4). These results indicate that the energy module has the potential to be used in real-world scenarios, including rural or low-resource settings where charging infrastructure is limited.

To validate clinical utility, we tested the insole during yoga. It distinguishes balance shifts between poses (e.g., warrior II and tree) with high accuracy, matching commercial pressure pads (Fig. S15). These results demonstrate performance comparable to commercial pressure mats, underscoring the system’s functionality in assessing balance control and movement precision during yoga practice. To demonstrate the system’s capability of dynamic activities, we further evaluated the smart insole’s performance during stair climbing and marching in place. Movies S5 and S6 illustrate the dynamic evolution of pressure distribution across 32 plantar regions on the left and right feet during stair climbing and marching in place, demonstrating the insole’s ability to perceive plantar pressure in real time. The insole consistently provided stable and accurate sensor signals, with heatmaps that closely matched the actual plantar pressure distribution.

By integrating high-efficiency PSCs with durable Li–S batteries, we overcome the energy autonomy challenge in wearable biomechanical monitoring. The system’s high photoelectric conversion efficiency and long-term runtime enable continuous gait analysis across real-world settings. This self-sustaining platform eliminates reliance on external charging, advancing telehealth applications such as remote prevention of foot pressure-related ulcers in patients with peripheral neuropathy and tracking of gait deterioration in individuals with osteoarthritis.

In consideration of environmental sustainability, the insole’s modular architecture facilitates partial recycling. The PEDOT/TPU composite and PDMS layers can be physically separated and reprocessed by solvent dissolution and thermal remolding, respectively. In addition, both the perovskite and Li–S energy components are designed for material recovery through standard recycling workflows, minimizing e-waste generation. Future optimization will emphasize the use of bio-based elastomers and green solvents to further enhance device sustainability.

### AI-assisted mechanodiagnosis of foot arch abnormalities and gait patterns using smart insole data

**Fig. 6.**
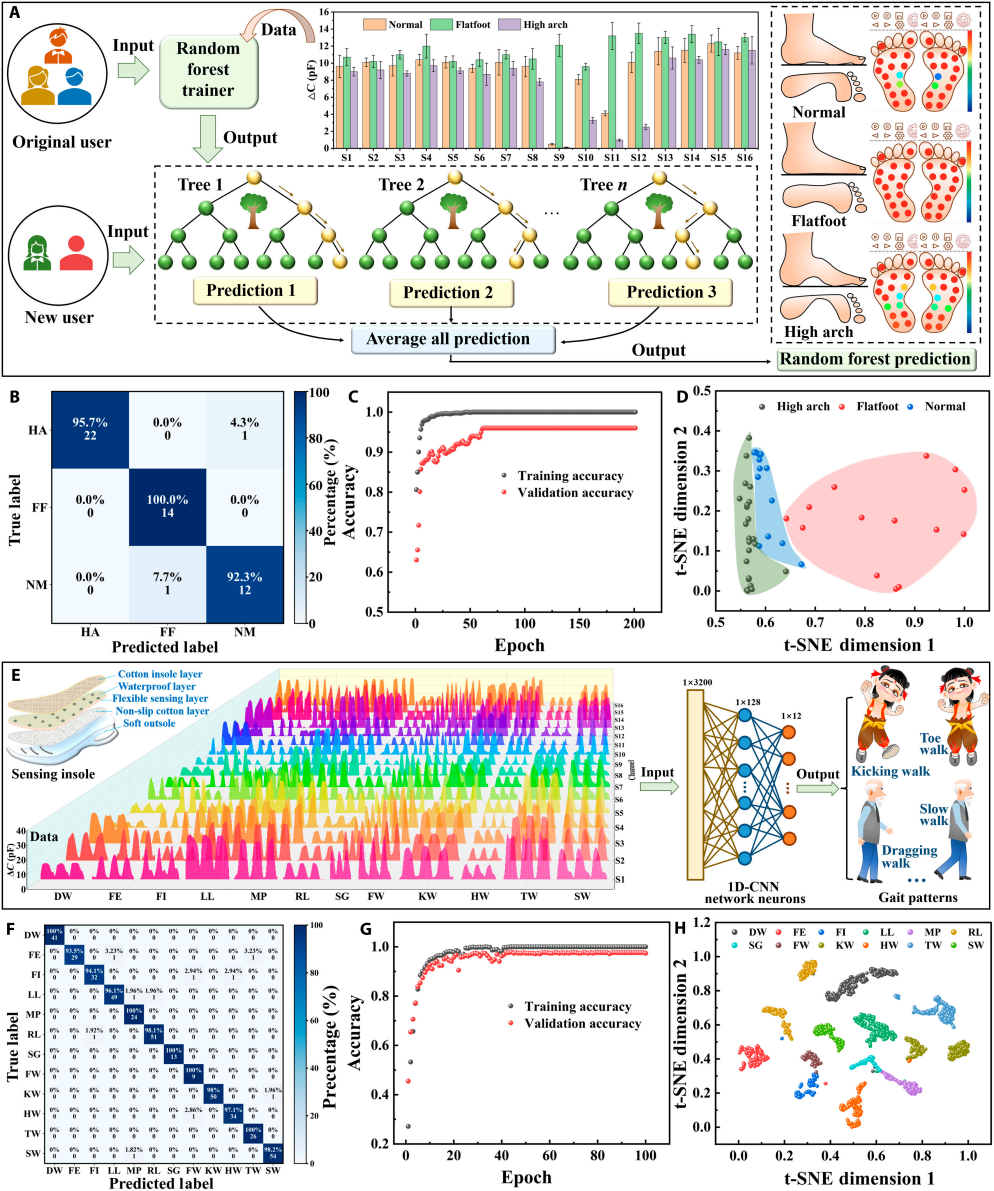
AI-assisted identification of arch diseases and gaits. (A) Bar graph of plantar pressure distribution and random forest algorithm for classifying normal, high-arched, and flat feet. (B) Confusion matrix result for the recognition of arch diseases. (C) Training and validation accuracy of random forest. (D) t-SNE visualization of the clustered data of the arch diseases. (E) Maps of the 16-channel dynamic data of 12 gait types and 1D-CNN framework. (F) Confusion matrix result for the recognition of 12 gaits. (G) Training and validation accuracy of 1D-CNN framework. (H) t-SNE visualization of the clustered data of gaits.

To enable automated, clinical-grade analysis of plantar pressure distribution, we integrated a machine learning framework with a 16-sensor smart insole system. A random forest model was trained on 500 static pressure profiles (35% high arch, 35% flatfoot, 30% normal) collected during upright standing, achieving 96% classification accuracy on an independent test set. The model identifies characteristic pressure deviations in flatfoot and high-arched feet, showing strong correlation with clinical diagnoses from podiatrists and laboratory force plates (Fig. 6A).

To demonstrate the analytical effects of AI, a color-coded confusion matrix (Fig. 6B) depicts high diagnostic precision, with pixel intensity representing classification confidence. We investigated the impact of readout layer size on recognition accuracy, revealing that reducing network size decreases accuracy, yet a ∼95% accuracy is maintained even at a network size of 65 (Fig. 6C). t-SNE (t-distributed stochastic neighbor embedding) visualization of multidimensional pressure data (Fig. 6D) revealed 3 distinct clusters corresponding to arch types, validating the model’s capacity to resolve subclinical biomechanical signatures for early intervention in structural foot abnormalities and stress fracture prevention.

To resolve gait time-dependent recognition issues, we engineered the 1D-CNN to analyze time-series capacitance data from 12 gait types, including slow walk (SW), fast walk (FW), marching in place (MP), left limp (LL), right limp (RL), shuffling gait (SG), dragging walk (DW), kicking walk (KW), toe walking (TW), heel walking (HW), foot eversion (FE), and foot inversion (FI). Figure 6E presents the capacitive signal outputs acquired by the smart insole during the identification of distinct gait patterns, alongside a schematic illustration of the 1D-CNN framework used for gait classification.

The gait recognition system based on 1D-CNN algorithm exhibits a classification accuracy of up to 97.6%, demonstrating excellent performance in distinguishing 12 pathological and physiological gait patterns (Fig. 6F). From the model training process, it can be observed that the model converges rapidly, achieving an accuracy of ~97% after around 45 epochs (Fig. 6G), indicating both efficient training and strong classification capability. Figure 6H further demonstrates the t-SNE dimensionality reduction outcomes, with well-defined clustering patterns for the 12 gait types, offering promising applications in the early diagnosis of diabetic neuropathy, osteoarthritis, and stroke-related gait asymmetry.

This AI-enhanced platform transforms raw plantar pressure data into clinical-grade diagnostics, detecting structural foot arch abnormalities and dynamic gait anomalies through biomimetic sensing-adaptive machine learning integration. The dual-modality system achieves 96% accuracy for static arch classification and 97.6% accuracy for 12 gait pattern recognition, surpassing the accuracies of other algorithms (Fig. 6F and Figs. S16 and S17), which demonstrates capabilities in early subclinical gait deviation detection (e.g., prodromal Parkinsonian gait) and personalized rehabilitation monitoring (e.g., asymmetric gait quantification).

Compared to the algorithm used in the paper, other algorithms have critical limitations: (a) For static arch disease identification, 1D-CNN models (Fig. S16) show an inadequate accuracy of about 80% (Δ = 16% versus random forest models) and premature training saturation for arch classification. Random forest was selected for arch-type classification owing to its robustness against overfitting, interpretability of feature importance, and superior performance with small, structured datasets derived from static plantar pressure features. (b) Transformer architectures (Fig. S17A) demand longer training time per epoch and require more epochs to reach a gait recognition accuracy of about 97.6%, indicating that the Transformer model demands higher computational power and time requirements. Additionally, due to the Transformer model’s lower efficiency in capturing local features, its stability is insufficient, as evidenced by Fig. S17B and C. 1D-CNN was employed for dynamic gait analysis because it automatically extracts hierarchical temporal features from continuous plantar pressure sequences, outperforming traditional handcrafted feature models in capturing spatiotemporal correlations.

In comparison with representative commercial smart insoles, our biomimetic smart insole exhibits marked advantages in multiple aspects. Its hierarchical bioinspired sensor achieves an ultralow detection limit of 0.10 Pa, a sensitivity of 0.602 kPa^−1^, and a broad dynamic range from 0.10 Pa to 1.40 MPa, outperforming commercial products that generally have lower sensitivity and narrower detection ranges. Moreover, our system supports energy-autonomous operation through integrated nano-PSCs and Li–S batteries, while most commercial devices rely on conventional rechargeable batteries. Finally, the embedded AI algorithms enable accurate foot arch abnormality detection (96.0%) and classification of 12 pathological gait patterns (97.6%), whereas existing commercial systems mostly provide raw pressure data or limited analytics. This comparison underscores the superior sensing performance, energy sustainability, and intelligent diagnostic capability of our insole, highlighting its potential for continuous gait monitoring, early disease screening, personalized rehabilitation, and remote healthcare.

## Conclusion

Overall, this work establishes a new paradigm for wearable biomedical devices by integrating biomimetic design, sustainable energy systems, and clinical-grade AI. The developed sensor achieves a low detection limit of 0.1 Pa, a broad sensing range of up to 1.4 MPa, and a high sensitivity of 0.602 kPa^−1^. The integrated system demonstrates an average photocharging efficiency of 11.21% and an energy storage efficiency of 72.15%. The AI-assisted gait recognition achieves 96.0% accuracy in detecting foot arch abnormalities and 97.6% accuracy in classifying 12 pathological gait patterns. By unifying real-time biomechanical data acquisition, chromatic force-field visualization, and explainable AI analytics, this work advances wearable technology from passive monitoring to actionable clinical decision-making. Its energy-independent design and scalability address critical barriers to adoption in underserved populations, where gait disorders are prevalent but underdiagnosed. As healthcare shifts toward preventive and personalized models, our closed-loop approach offers a scalable blueprint for elevating wearables to the status of essential medical devices. Future efforts will focus on large-scale longitudinal validation of fall-risk prediction and rehabilitation tracking, as well as integration with digital health ecosystems for predictive care.

## Materials and Methods

### Material

TPU foam was obtained from Suzhou Shensai New Materials Co. Ltd. Ferric chloride (FeCl_3_) and ethanol were purchased from Tianjin Damao Chemical Reagent Co. Ltd. PDMS was obtained from Dow Corning Co. Ltd. 3,4-Ethylene dioxythiophene (EDOT) and methanol were purchased from Sigma-Aldrich Chemical Co. Ltd.

### Preparation of PEDOT/TPU foam

To prepare the oxidant solution, 0.2 g of FeCl_3_ was mixed with 4 ml of methanol and stirred. The TPU foam was cleaned with anhydrous ethanol and deionized water, and then soaked in the oxidant solution for 15 min. After removing and heating at 70 °C for 15 min, a device for VPP was set up, fixing the TPU foam on a petri dish lid and dripping EDOT monomer onto the bottom filter paper. The device was heated at 75 °C for 2 h, flipping the foam after 1 h for even polymerization. The TPU/PEDOT foam was then cleaned with anhydrous ethanol and deionized water to remove excess monomers, oxidant solution, and other impurities.

### Preparation of the dielectric layer with micropyramid array

PDMS precursor and curing agent were mixed 10:1 and spin-coated on a photolithography silicon template with a micropyramid pattern at 1,000 rpm for 60 s. TPU/PEDOT foam was adhered to the PDMS and cured at 80 °C for 3 h. Single-sided and double-sided micropyramid array PEDOT/TPU foams were prepared in turn using the aforementioned method. Electrodes and a dielectric layer were assembled using copper foil and the micropyramid array PEDOT/TPU foam, encapsulated with breathable 3M Tegaderm tape.

### Characterization and measurement of the capacitive pressure sensor

FTIR (NEXUS870, Thermo Nicolet) and Raman spectrometer (Xplora plus, HORIBA) were used to analyze chemical bonds and chemical structures of the composite materials. SEM (S-570, Hitachi) was applied to characterize the morphology of the materials. The mechanical and electrical properties of the sensors were analyzed simultaneously by a universal testing machine (3365, Instron) and a precision LCR meter (TruEbox-01RC, LinkZill). This LCR meter was miniaturized and portable, and featured a wireless connection.

### Cell viability and staining assay

Cell viability was assessed using CCK-8 cell viability assay kit (Beyotime, Shanghai, China) with L-929 cells seeded in 96-well plates at a density of 2 × 10^3^ cells per well and incubated with material extract. Optical density was measured at 450 nm using a microplate reader (Biotek, Burlington, VT, USA) after 1, 2, and 3 d. Acridine orange (AO) and ethidium bromide (EB) stained live and dead cells, respectively, in L-929 cells (a density of 2 × 10^5^ cells per well), which were cultivated in 6-well plates with material extract for 1, 2, and 3 d, observed under a Nikon fluorescence microscope (Japan, Ti-S).

### Solar cell fabrication and characterization

The PSCs were deposited on the indium tin oxide (ITO) glass substrates (2 × 2.5 cm^2^, 10 Ω per square). The cleaned ITO substrates were treated by ultraviolet (UV)-ozone for 20 min. Then, the tin oxide (SnO_2_) film was deposited on the ITO substrate by spin-coating the SnO_2_ precursor (5%) at 4,000 rpm for 30 s and annealed on a hot plate at 150 °C for 30 min in air. After cooling down to room temperature, the substrate was treated with UV-ozone for 10 min before being transferred to a nitrogen-filled glove box. For the perovskite film preparation, 691.5 mg of lead (II) iodide (PbI_2_) and 19.5 mg of cesium iodide (CsI) were dissolved in 900 μl of *N*,*N*-dimethylformamide, with 144 μl of *N*-methylpyrrolidone added and stirred for 2 h at 70 °C. Formamidinium iodide (FAI) (90 mg) and methylamine hydrochloride (MACl) (14 mg) were dissolved in 1 ml of isopropanol (IPA) and stirred for 30 min at room temperature. Then, 75 μl of the mixed PbI_2_ and CsI solution was spin-coated onto prepared SnO_2_ at 2,000 rpm for 30 s followed by 1 min of annealing at 70 °C. Then, 200 μl of FAI:MACl solution was spin-coated onto the prepared film at 4,000 rpm for 30 s, followed by thermal annealing at 150 °C for 15 min under N_2_ atmosphere. 2,2′,7,7′-Tetrakis[*N*,*N*-di(4-methoxyphenyl)amino]-9,9′-spirobifluorene (Spiro-OMeTAD) solution was spin-coated on the top of the perovskite layer at 4,000 rpm for 45 s, which contained 72.5 mg of Spiro-OMeTAD, 17.5 μl of bis(trifluoromethane) sulfonimide lithium salt (Li-TFSI) solution (520 mg/ml in acetonitrile), 28.8 μl of tris(2-(1*H*-pyrazol-1-yl)-4-tert-butylpyridine)-cobalt(III)tris(bis(trifluoromethylsulfonyl)imide)) (FK209) solution (300 mg/ml in acetonitrile), and 28.8 μl of 4-tert-butylpyridine (tBP) in 1 ml of chlorobenzene (CB). Finally, a 100-nm-thick layer of Ag was thermally evaporated on the top of Spiro-OMeTAD as electrodes. To evaluate PSC performance under sunlight, it was illuminated by a normalized AM1.5G solar simulator (100 mW·cm^−2^). PSC photoelectric properties were tested using photovoltaic cell testing equipment.

### Fabrication of smart flexible insole system

The electrode and circuit of the insole were printed on the flexible polyimide substrate. A 16-channel capacitive pressure sensor array was assembled and strategically placed across the heel, midfoot, and forefoot areas of the insole. The pressure sensors were packaged and connected to an FPCB using flat cables. The microcontroller unit (MCU) of the FPCB, which was based on the STM32 main controller chip, was capable of collecting capacitive signals from the 16-channel pressure sensors. The pressure data were then wirelessly transmitted to a mobile APP terminal through the Bluetooth chip embedded in the FPCB. The sensing module transmitted data to the mobile APP in real time via Bluetooth 5.0 at a sampling rate of 25 Hz. All human subject experiments were conducted with the voluntary and informed consent of the participants, with written consent obtained prior to any demonstrations involving human skin.

### Static foot arch abnormality detection and dynamic gait pattern recognition

This study was conducted with approval from the Medical Ethics Committee of the Northwest University (250120003). It focuses on recognizing static foot arch types by constructing a classification model based on plantar capacitance data collected through smart insoles. During the experiment, participants wore smart insoles and maintained a static standing posture for a period of time. In the data collection process, a total of 500 valid samples were collected, consisting of 35% high arches, 35% flat feet, and 30% normal arches. The dataset was randomly split into 90% training set and 10% test set. Based on the train set, the random forest algorithm was used to train the model, and after parameter tuning, the optimal classifier was obtained. Finally, the model’s performance was validated and evaluated using the test set, with key metrics such as accuracy and confusion matrix calculated to assess its classification performance and generalization ability.

This study also addressed the challenge of dynamic gait identification. Participants wore smart insoles and walked for a period of time with a specific gait, generating a dataset consisting of 2,110 samples, each containing 16 × 200 capacitance signals. The dataset was split into 80% training set and 20% test set randomly. A 1D-CNN framework was used to train the model for 100 epochs using the training set, and the model’s performance was evaluated using the test set after per epoch. Key metrics, including loss, accuracy, and the confusion matrix, were used to assess the model’s performance and generalization ability. Additionally, analyzing the loss rate and accuracy curves for training set and test set could identify the most effective training epoch or epoch range, reducing overfitting, indicating that the model could generalize well to the unseen dataset. The analysis aimed to evaluate the effectiveness of the model in identifying different dynamic gaits and its potential for analysis in medical diagnosis.

## Data Availability

The data supporting the findings of this study are available within this article and its Supplementary Materials.
