# Supplementary material for: Mantis Leg-Inspired Smart Insole Integrating Closed-Loop Power Supply for Advanced Wearable Gait Diagnostics
Source: Research (Wash D C). 2026 Jan 8;9:1063. doi: 10.34133/research.1063 (PMC12779891; doi:10.34133/research.1063)
Supplement: Supplementary 1 — Figs. S1 to S17 Table S1 Movies S1 to S6 [file research.1063.f1.zip › Supporting Information.docx]

**Mantis-Leg-Inspired Smart Insole Integrating Closed-Loop Power Supply for Advanced Wearable Gait Diagnostics**

Yingchun Li^1,#^, Yarong Ding^2^^,#^, Yuze Zhang^3,#^, Xing Guo^1^, Kaixin Lei^3^, Jiachun Sun^1^, Xing Hu^2^, Xinyue Li^2^, Wenguang Yang^2^, Rui Liu^4^, Zhenhua Lin^2^, Wendong Zhang^4^, Shaozhe Tan^2^, Xu Yang^5^, Yumeng Xu^2^, Jin Tian^6,7^, Bokun Zhang^6,7^, Yue Hao^2^, Xiangning Li^3,*^, Yannan Liu^5,*^, Feng Xu^6,7*^ and Jingjing Chang^1,2*^

*^1^* *Advanced Interdisciplinary Research Center for Flexible Electronics, Academy of Advanced Interdisciplinary Research, Xidian University, Xi’an 710071, P.R. China*

*^2^ State Key Laboratory of Wide-Bandgap Semiconductor Devices and Integrated Technology,* *Faculty of Integrated Circuit, Xidian University, Xi’an 710071, P.R. China*

*^3^ Institute of Electromechanical Science and Technology, Xidian University, Xi’an 710071, P.R. China*

*^4^ Department of Rehabilitation Medicine, Tangdu Hospital, Air Force Military Medical University, Xi’an, 710032, P.R. China*

*^5^ Shaanxi Key Laboratory of Degradable Biomedical Materials, School of Chemical Engineering, Northwest University, Xi’an 710069, P.R. China*

*^6^ The Key Laboratory of Biomedical Information Engineering of Ministry of Education, School of Life Science and Technology, Xi’an Jiaotong University, Xi’an 710049, P.R. China*

*^7^ Bioinspired Engineering and Biomechanics Center (BEBC), Xi’an Jiaotong University, Xi’an 710049, P.R. China*

*^#^ These authors contributed equally to this work.*

*^*^ Corresponding authors: jjingchang@xidian.edu.cn; fengxu@mail.xjtu.edu.cn;* [*liuyannan@nwu.edu.cn*](mailto:liuyannan@nwu.edu.cn)*; lixn@xidian.edu.cn*

**Supplementary Legend for Videos:**

**Video S1.** FEA simulation of the sensor with double-sided pyramid microstructure.

**Video S2.** The signal stability of the pressure sensor affixed to the tire tread of a running car.

**Video S3.** LEDs powered by PSC series, forming various lighting patterns.

**Video S4.** Smart insole powered by both Li-S batteries and PSCs.

**Video S5.** Real-time gait monitoring during walking up the stairs.

**Video S6.** Real-time gait monitoring during marching in place.

**Supplementary Figures:**


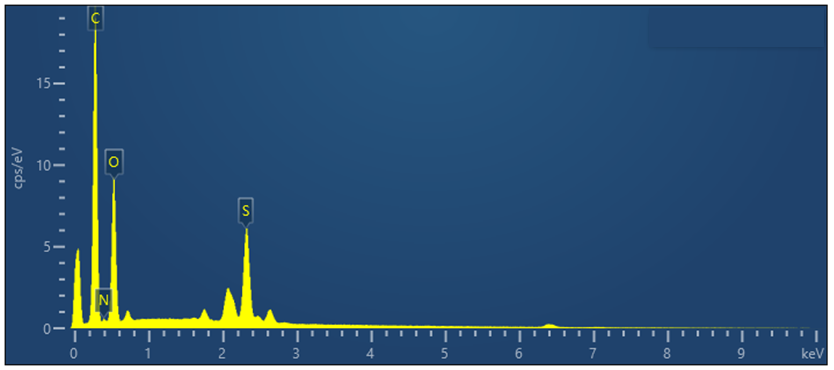


**Fig.** **S1** Energy dispersive spectroscopy (EDS) mapping of C, N, O, S element on the surface of PEDOT/TPU foam.


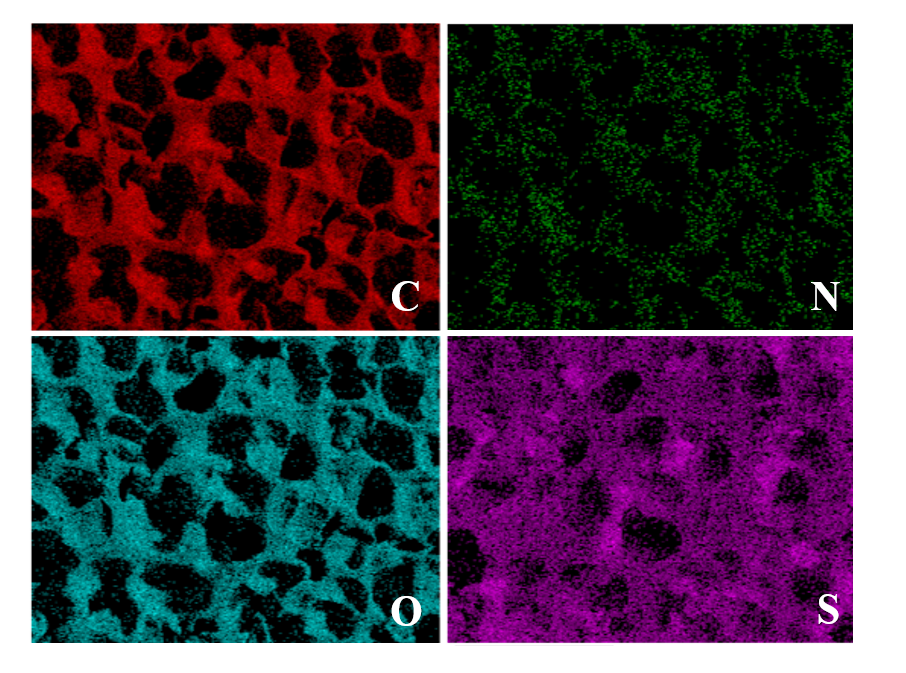


**Fig. S2** Mappings of C, N, O, S element on the surface of PEDOT/TPU foam.


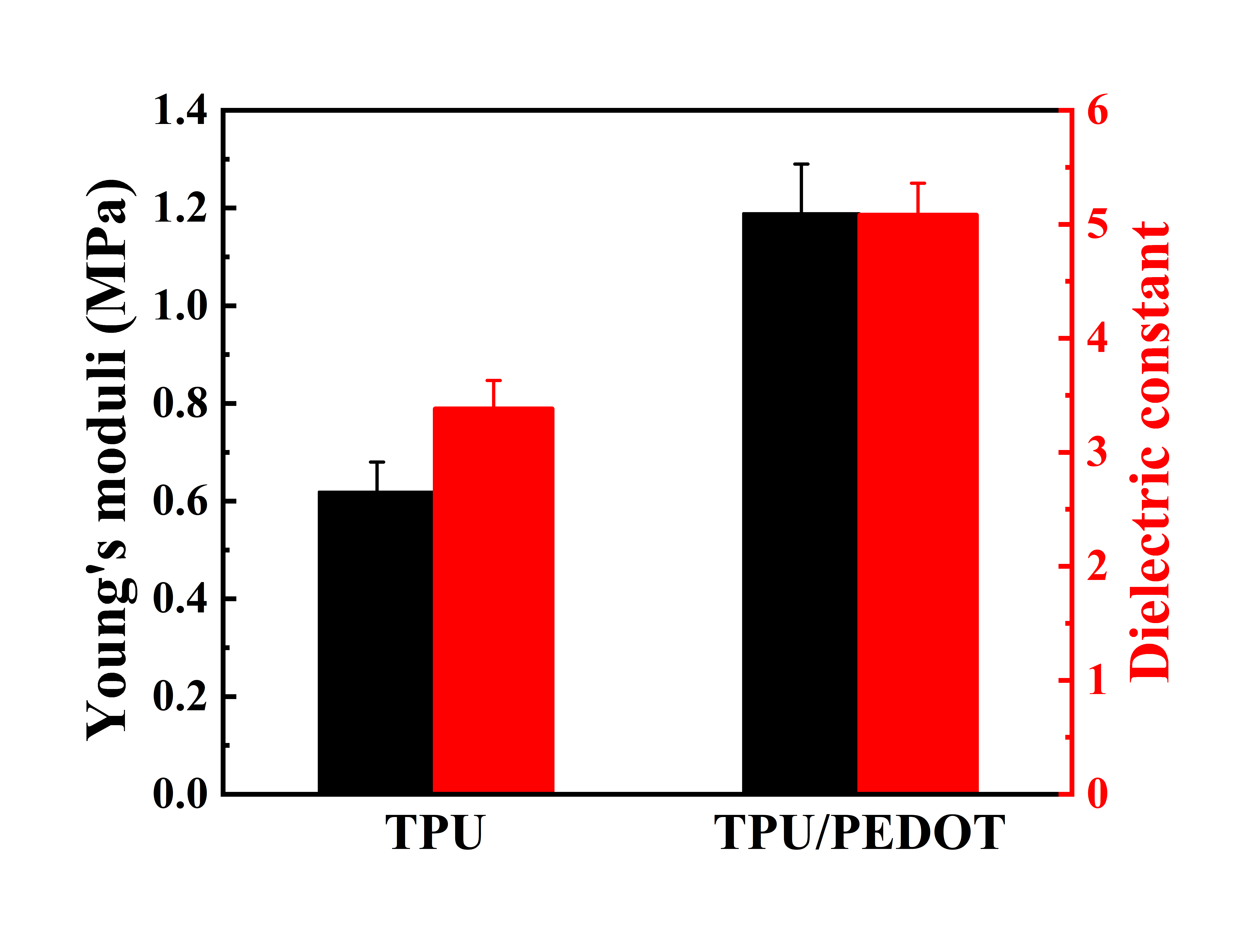


**Fig. S3** Young's modulus and dielectric constant of TPU and TPU/PEDOT foam.


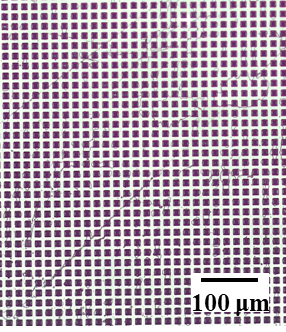


**Fig. S4** Optical images of the micropyramid array of the dielectric layer.


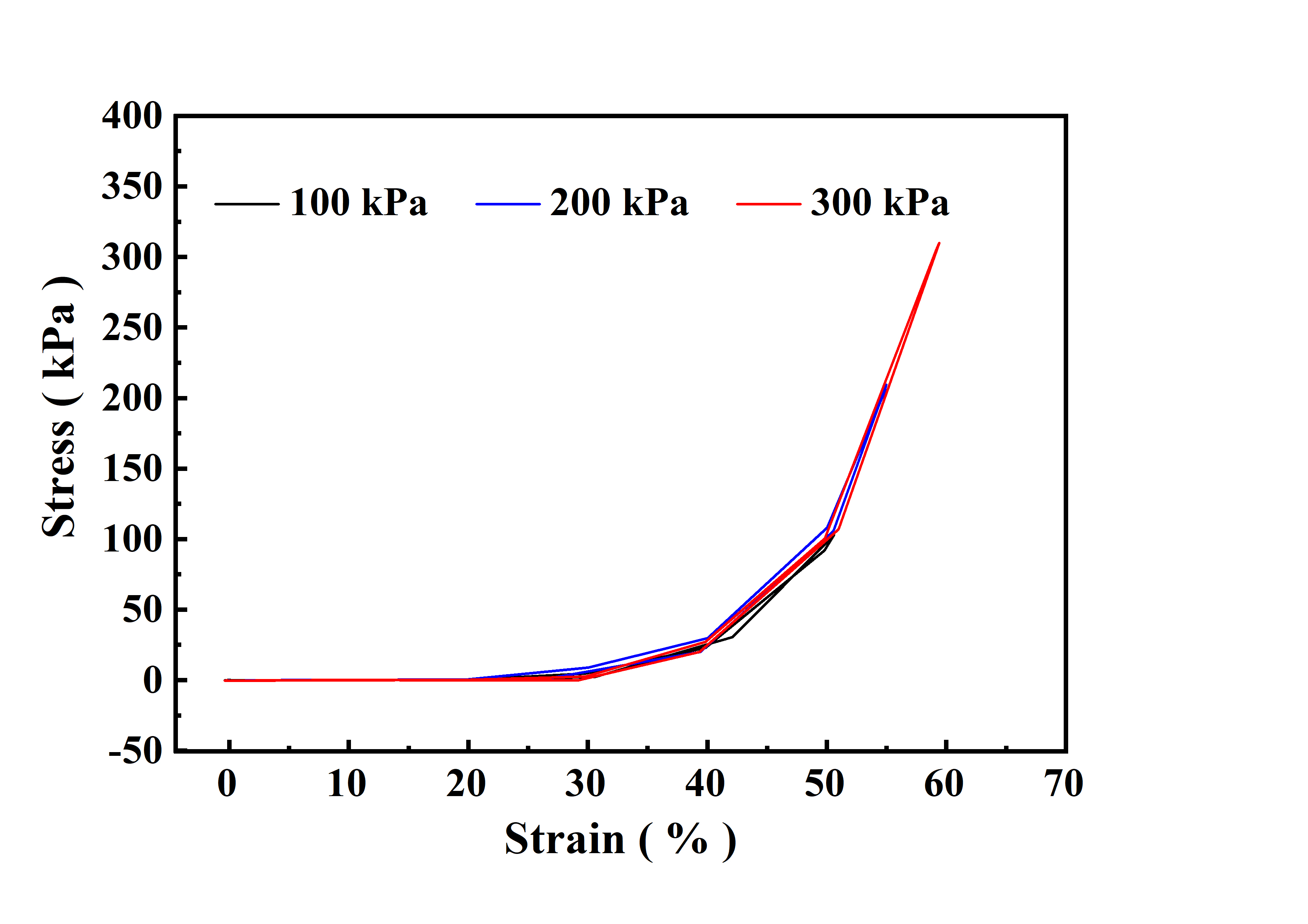


**Fig. S5** Stress-strain curves of the sensor with high pressure loading.


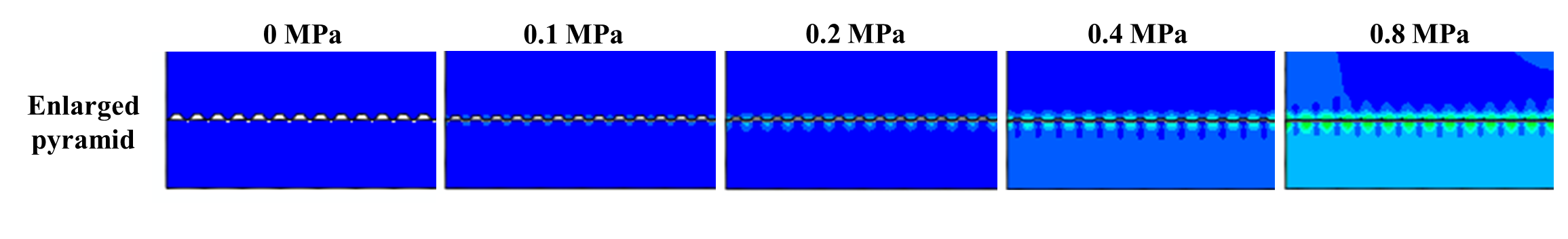


**Fig. S6** The enlarged image of finite element simulation analysis of the pressure sensor with double-sided pyramid microstructures during the pressing process.


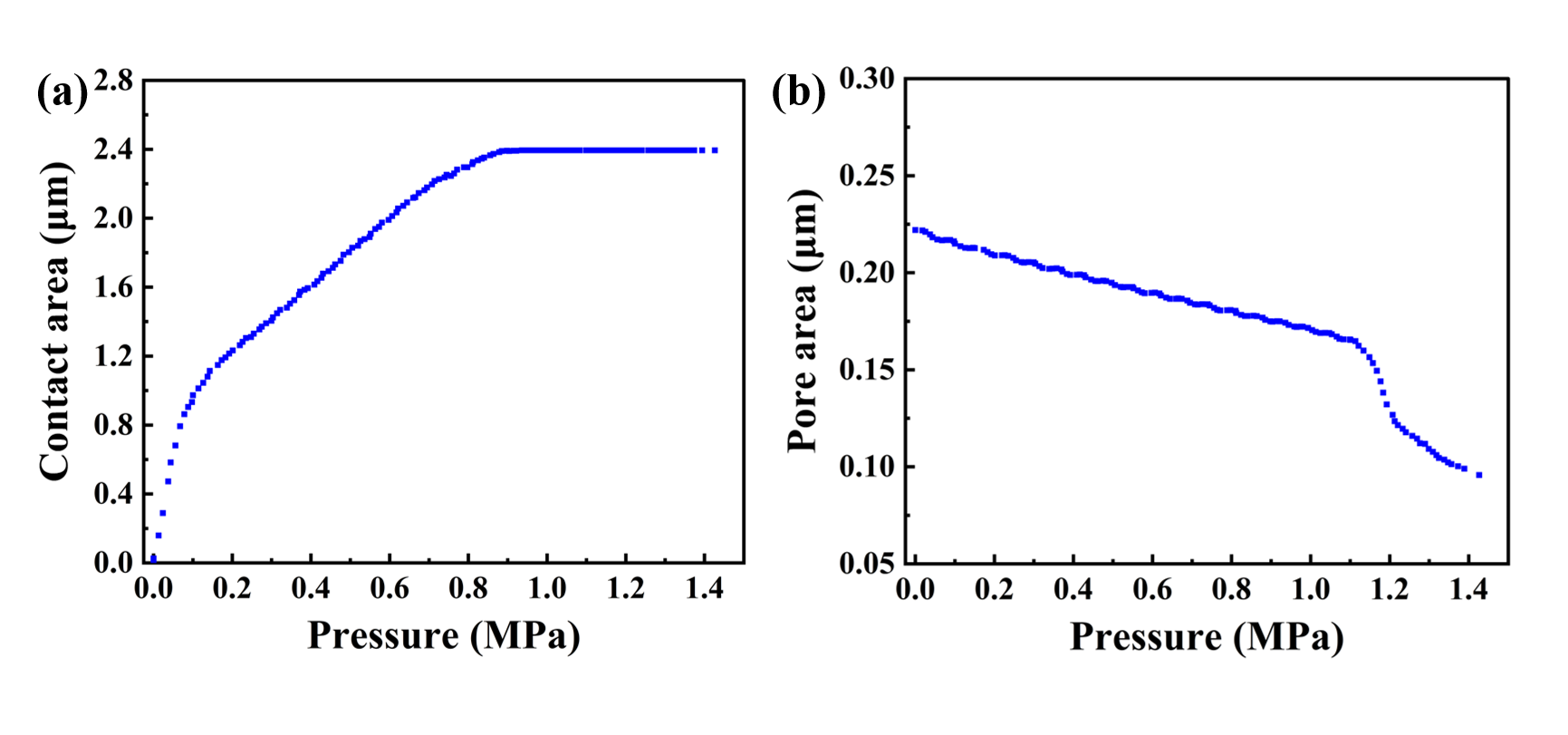


**Fig. S7** (a) The simulation curves of the pyramid contact area during pressure loading. (b) The simulation curves of the pore area in the TPU foam during pressure loading.


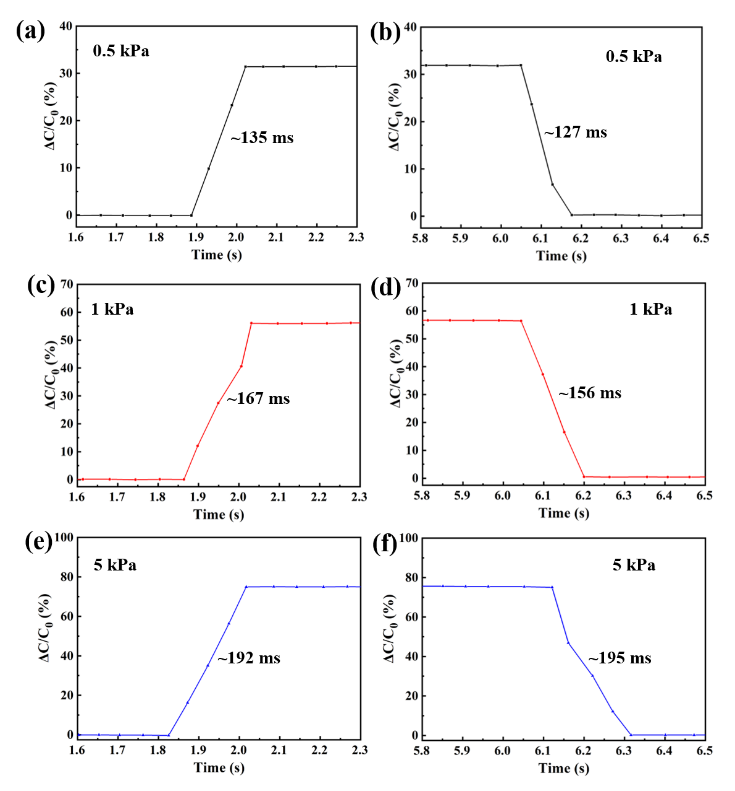


**Fig. S8** Response times (a, c, e) and recovery times (b, d, f) at three pressure levels (0.5, 1, and 5 kPa).

**
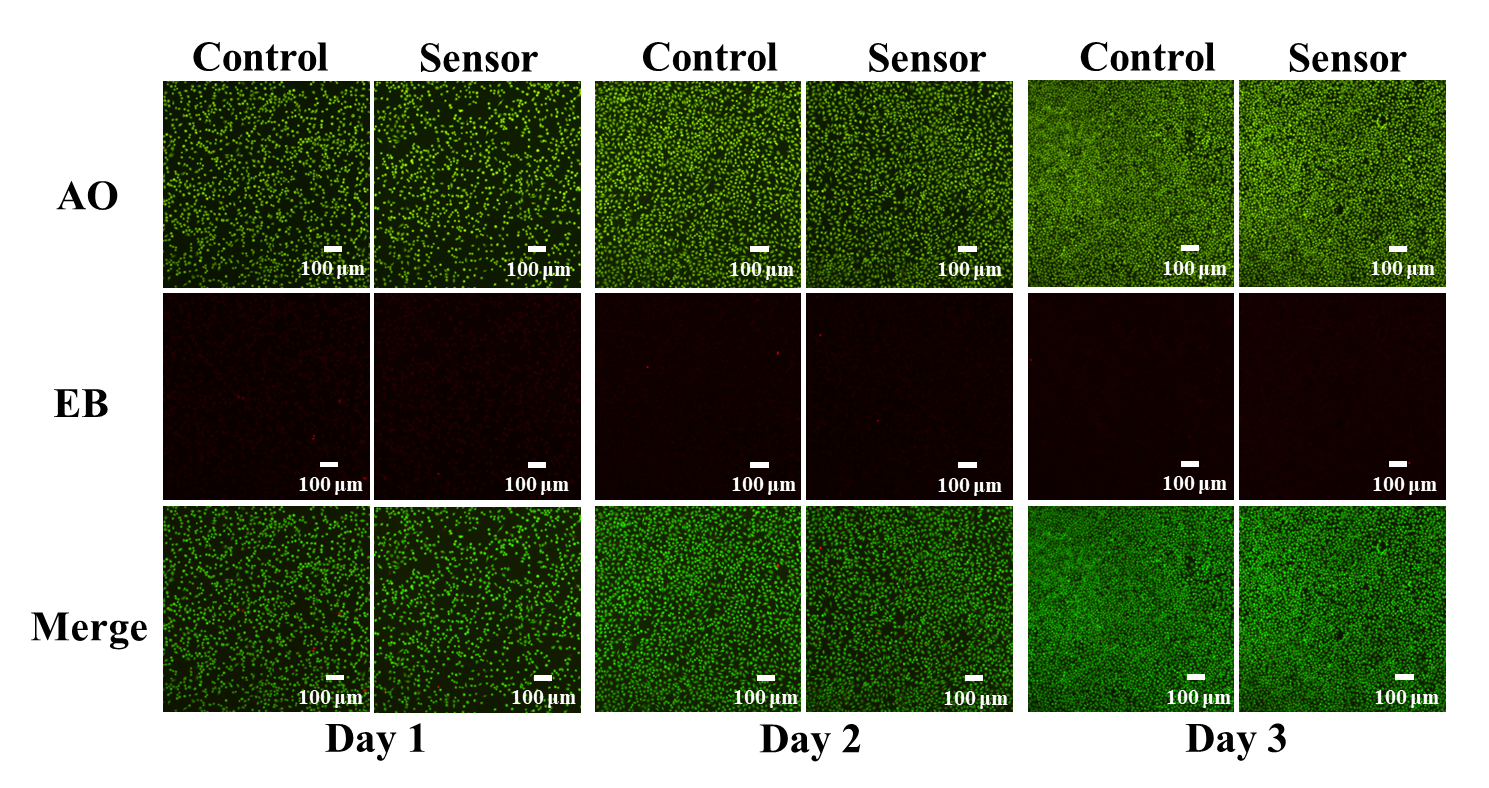
**

**Fig. S9**  Live/dead staining of L929 cells incubated with sensors extract for 1, 2 and 3 days.


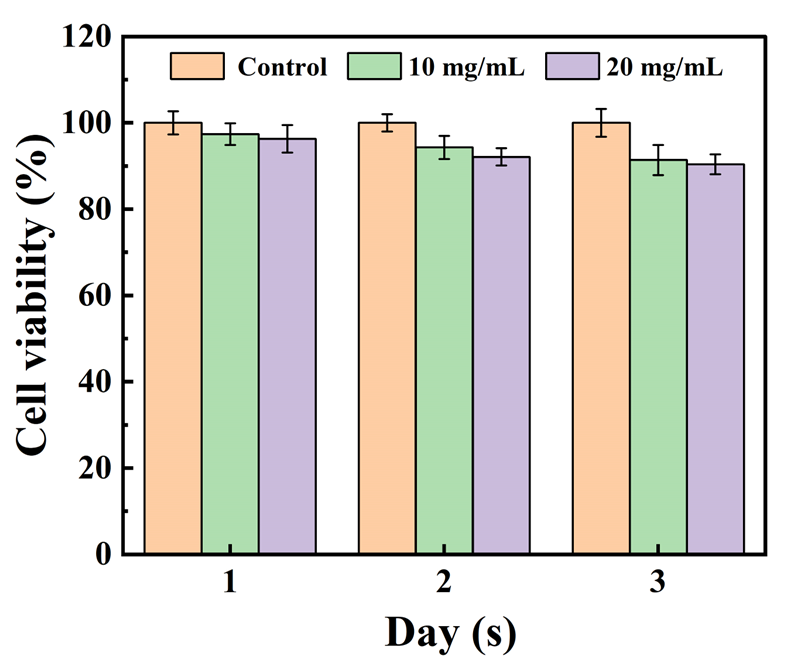


**Fig. S10** Cell survival efficiencies of sensors extract for 1, 2 and 3 days.


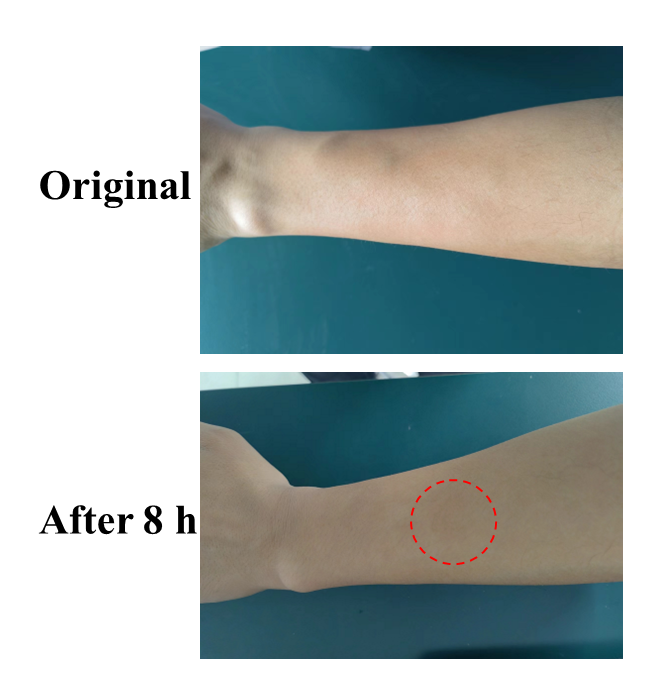


**Fig. S11** Photographs of the sensors attached to human skin before and after 8 h.


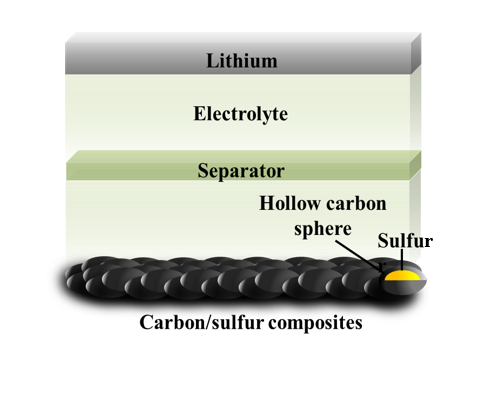


**Fig. S12** Schematic illustration of the device structure of Li-S battery.


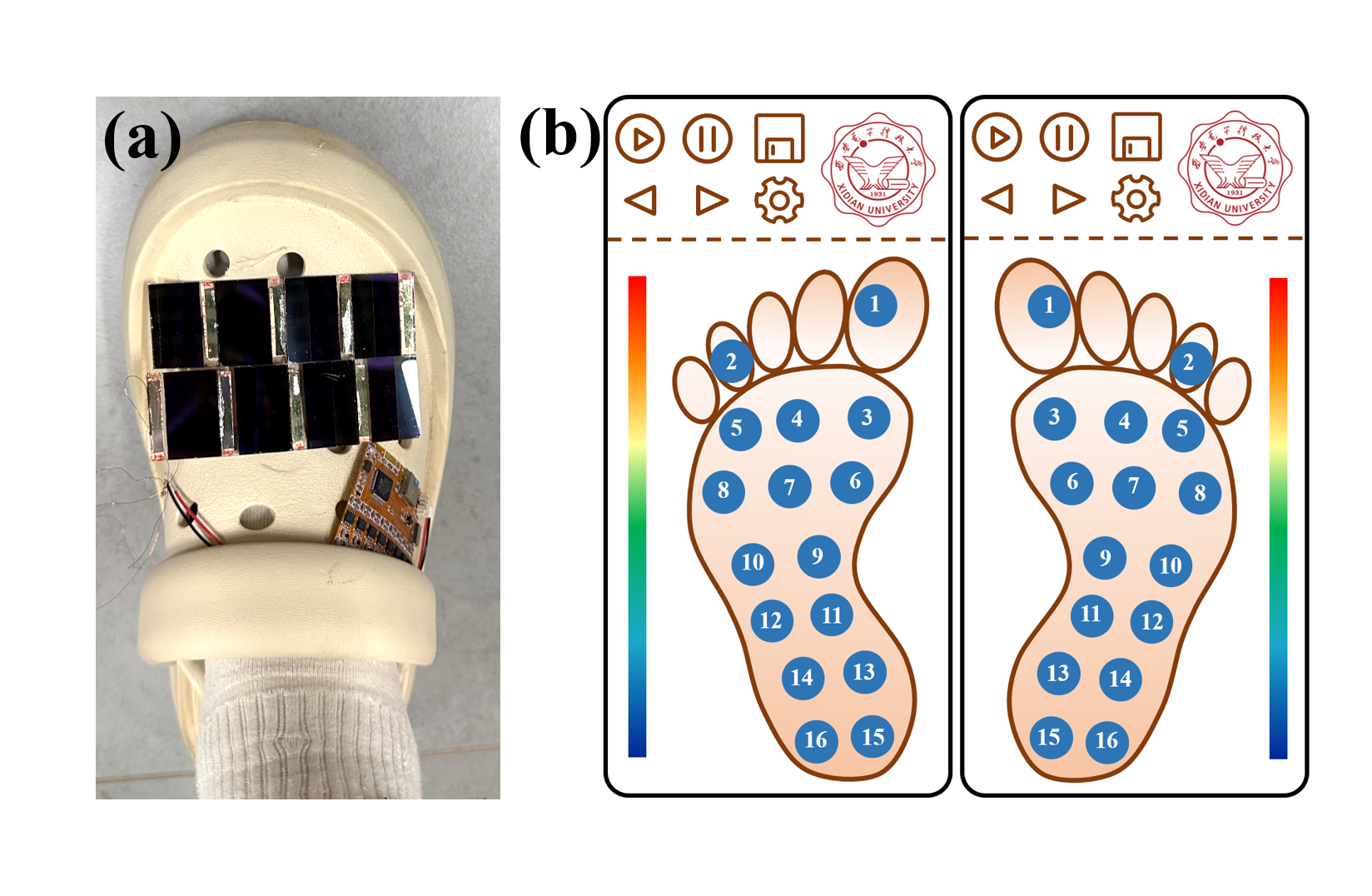


**Fig. S13** (a) FPCB powered by PSC series. (b) Photos of APP interface.


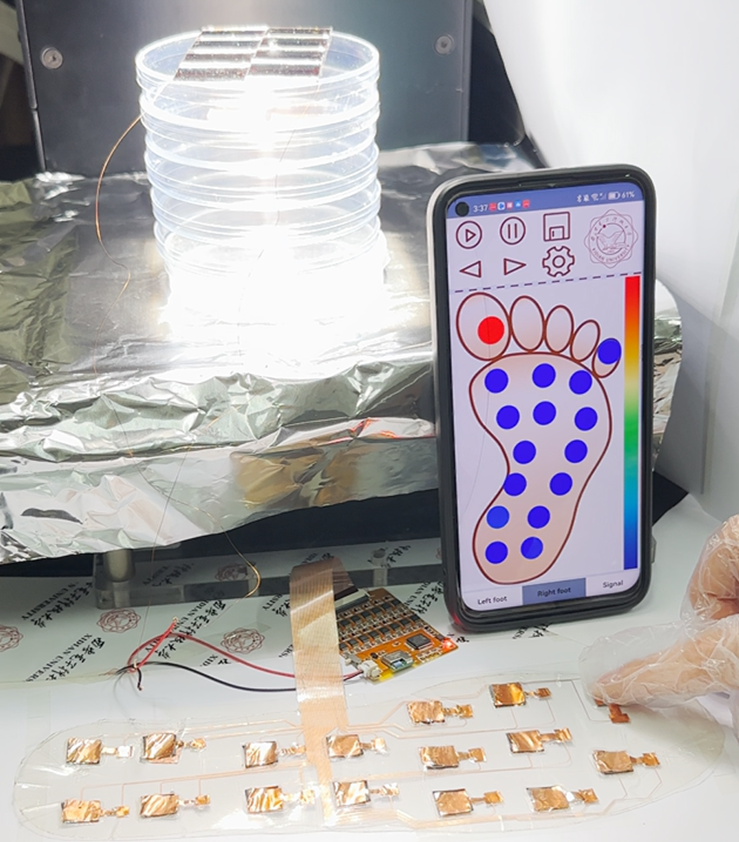


**Fig. S14** Control circuit powered by PSCs and displayed on cellphone.


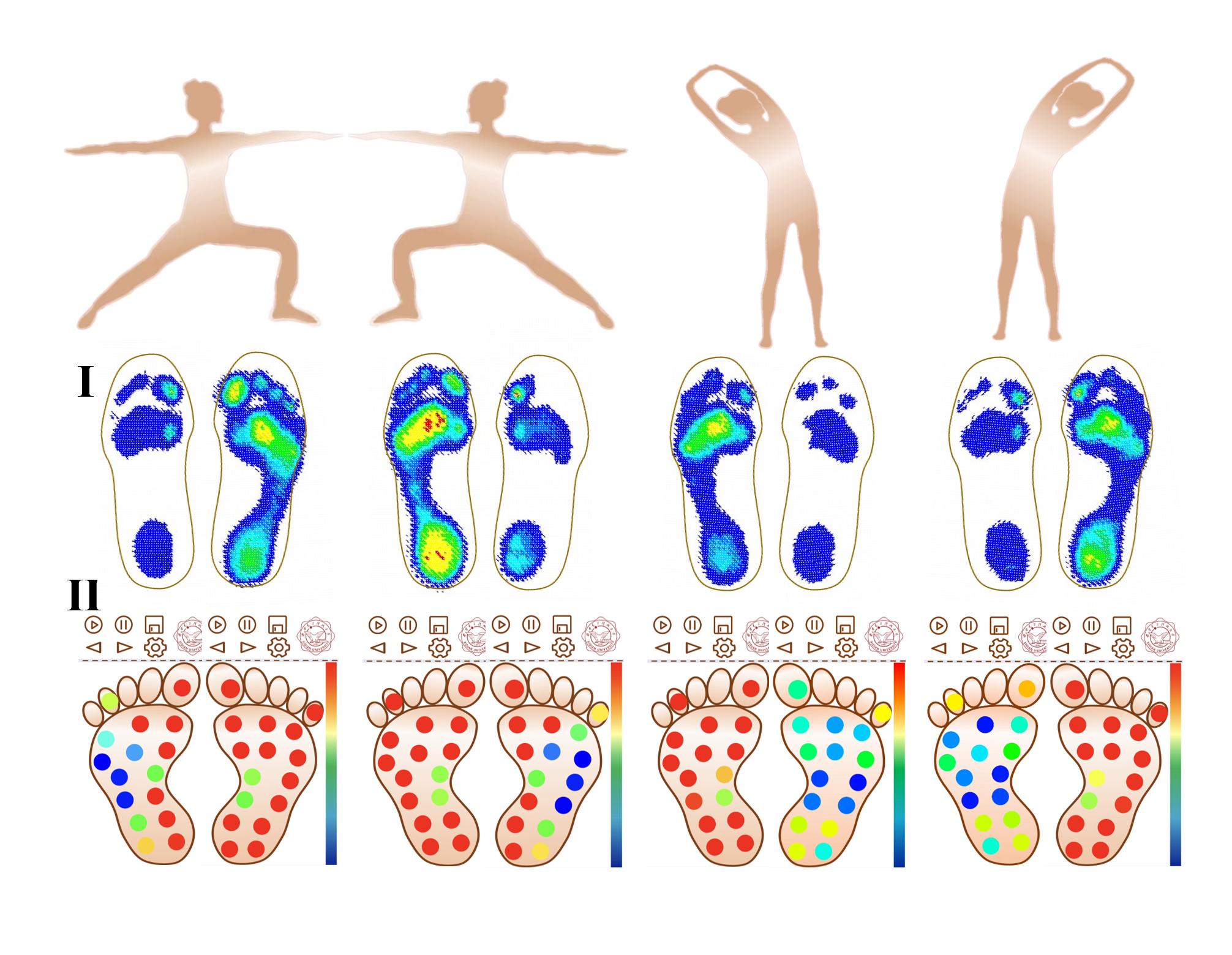


**Fig. S15** Smart insole for yoga gait monitoring with poses schematic. (I-II) Plantar recognition using commercial pad and smart insole.


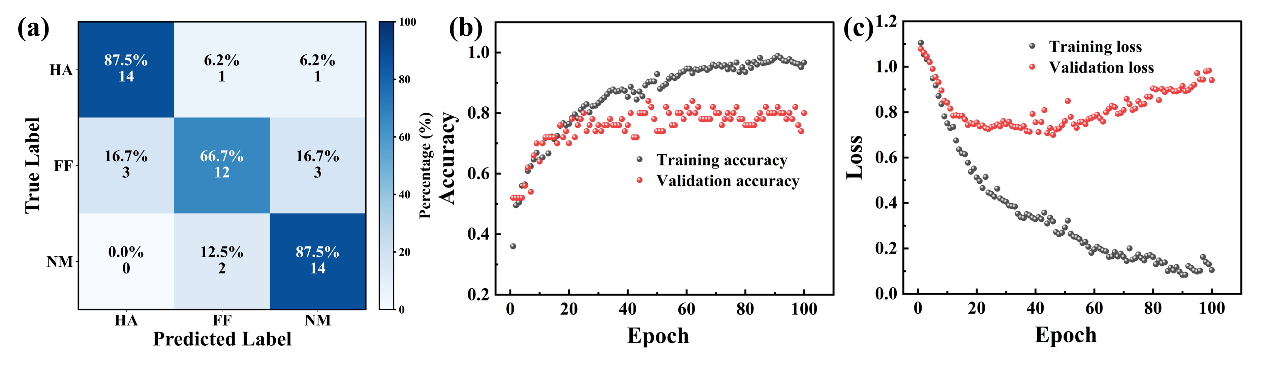


**Fig. S16** (a) Confusion matrix results for static arch disease identification using the 1D-CNN framework. (b) Training and validation losses of 1D-CNN framework for static arch disease identification. (c) Training and validation accuracies of 1D-CNN framework for static arch disease identification.


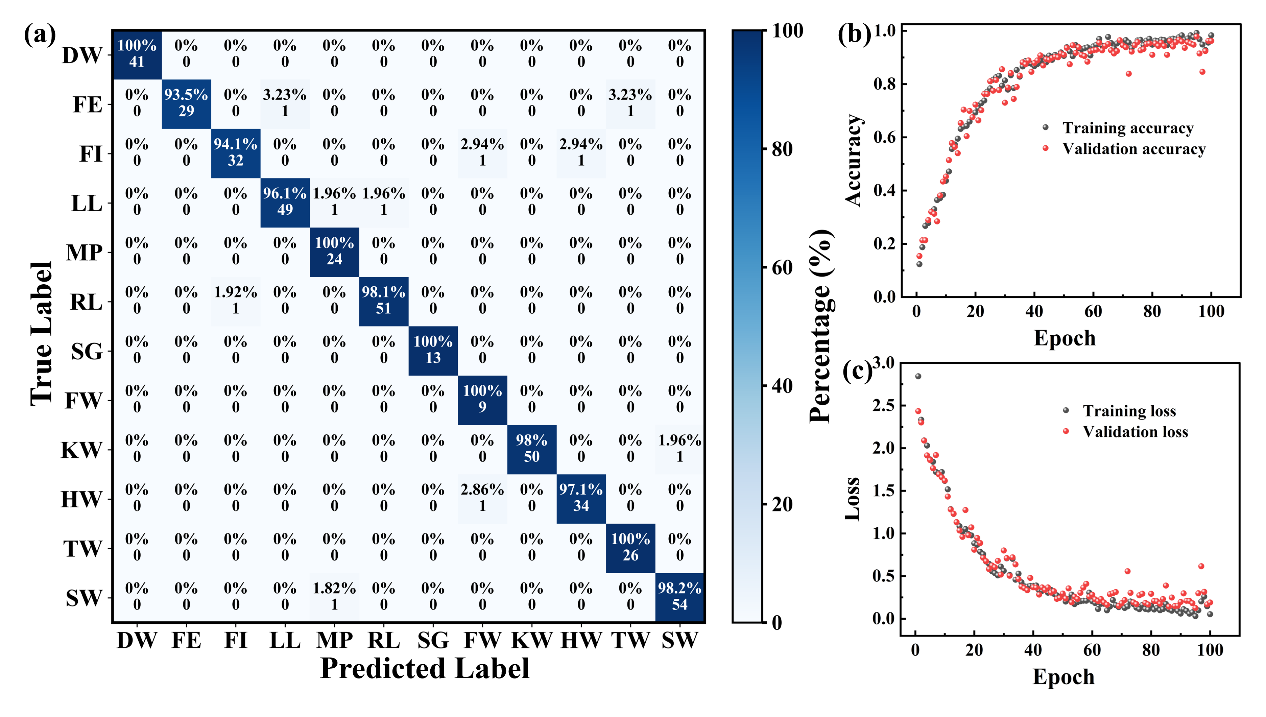


**Fig. S17** (a) Confusion matrix results for the recognition of 12 gaits using the Transformer framework. (b) Training and validation losses of Transform framework for the recognition of 12 gaits. (c) Training and validation accuracies of Transform framework for the recognition of 12 gaits.

**Supplementary Table:**

**Table S1** Comparison of sensing performance between various design of capacitive pressure sensors.

| Dielectric material | Electrode material | Sensitivity | Detection range | Response time/ Recovery time | Ref. |
| --- | --- | --- | --- | --- | --- |
| PDMS/CNTs | PDMS/CNTs | 0.15 kPa^-1^ | 450 kPa | 6 ms/6 ms | [1] |
| PDMS/BaTiO_3_ | AgNWS-LOOP@PET | 0.005 kPa^-1^ | 100 kPa | 70 ms/- | [2] |
| Poly (glycerol sebacate) | Mg/Polylactic acid | 0.13 kPa^-1^ | 10 kPa | 150 ms/150 ms | [3] |
| Graphene nanosheets | Cu/Ni | 0.33 kPa^-1^ | 110 kPa | 50 ms/110 ms | [4] |
| PDMS | PDMS/Cu | 0.22 kPa^-1^ | 500 kPa | 4.2 ms/5.3 ms | [5] |
| PDMS | P(TFEA-co-AAm)-[EMIM][TFSI] ionogel | 0.51 kPa^-1^ | 1000 kPa | 100 ms/200 ms | [6] |
| CIP/NdFeB/PDMS | CNT/PDMS | 0.314 kPa^-1^ | 1000 kPa | 200 ms/300 ms | [7] |
| CB-PVDF-HFP/ [EMIM][TFSI] | ITO/PET | 0.35 kPa^-1^ | 250 kPa | 110 ms/220 ms | [8] |
| PVDF/AgNWs/TiO_2_ | Au | 0.0012 kPa^-1^ | 1500 kPa | 166.9 ms/199 ms | [9] |
| PDMS | Gallium/Liquid metal | 0.007 kPa^-1^ | 100 kPa | 80 ms/80 ms | [10] |
| TPU/EMIS:TFSI | PEDOT:PSS | 0.5 kPa^-1^ | 400 kPa | - | [11] |
| Cotton fiber/PU | Cu | 0.0024 kPa^-1^ | 500 kPa | - | [12] |
| Ion-gel | AgNWs/PEDOT | 0.32 kPa^-1^ | 50 kPa | 227 ms/232 ms | [13] |
| Nanofiber/PDMS | CNT/PDMS/MXene | 0.091 kPa^-1^ | 2000 kPa | 71 ms/117 ms | [14] |
| EPU | Cu | 0.0063 kPa^-1^ | 400 kPa | 1.1 s/2.58 s | [15] |
| FEC/ACMO/ AM | ITO | - | 1080 kPa | - | [16] |
| Pectin xerogel | Carbon paste coated cellulose film | 0.0294 kPa^-1^ | 100 kPa | 118 ms/130 ms | [17] |
| MWNTs/PDMS | CNT-Ecoflex | 0.12 kPa^-1^ | 130 kPa | 46 ms/44 ms | [18] |
| PVDF/HFP/μNi | Cu | 0.0982 kPa^-1^ | 100 kPa | 24 ms/24 ms | [19] |
| PVDF/rGO | Ag | 0.18 kPa^-1^ | 360 kPa | 56.3 ms/- | [20] |
| PHB/PHV | CNT/PU | 0.19 kPa^-1^ | 680 kPa | - | [21] |
| Cotton fabric | Ag | 0.036 kPa^-1^ | 110 kPa | 104 ms/96 ms | [22] |
| PDMS | Au/PET | 0.139 kPa^-1^ | 45 kPa | - | [23] |
| PDMS/PVA | Cu | 0.43 kPa^-1^ | 120 kPa | - | [24] |
| PDMS | AgNWs/MXene | 0.075 kPa^-1^ | 30 kPa | 65 ms/- | [25] |
| TPU/PEDOT/PDMS | Cu | 0.602 kPa^-1^ | 1400 kPa | 135 ms/127 ms | Our work |

ITO: Indium tin oxide

PET: Poly(ethylene terephthalate)

MWCNTs: Multi-walled carbon nanotubes

MTMPA: Magnetic tilted micropillar array

PEDOT:PSS: Poly(3,4-ethylenedioxythiophene)-poly(styrenesulfonate)

IG：Ionic gel

EPU: elastomeric polyurethane

FEC: fluorinated ethylene carbonate

ACMO: 4-acryloyl morpholine

AM: acrylamide

PHB: polyhydroxybutyrate

PHV: polyhydroxyvalerate

PVDF: poly(vinylidene fluoride)

HFP: hexafluoropropylene

Carbonyl iron powder/NdFeB/PDMS (CIP/NdFeB/PDMS)

Carbon black (CB)-embedded poly(vinylidene fluoride-co-hexafluoropropylene) (PVDF-HFP)/ [EMIM][TFSI]

Polyvinylidene fluoride (PVDF)@AgNWs@TiO_2_

**References:**

1. Zhang Y, Yang J, Hou X *et al.* *Nat. Commun.* 2022; **13**(1): 1317.

2. Luo Y, Chen X, Li X *et al.* *Nano. Res.* 2023; **16**(5): 7550-7558.

3. Boutry CM, Kaizawa Y, Schroeder BC *et al.* *Nat. Electron.* 2018; **1**(5): 314-321.

4. Ye X, Shi B, Li M *et al.* *Nano Energy*. 2022; **97**: 107114.

5. Ji Y, Zhang Y, Zhu J *et al.* *Small*. 2023; **19**(24): 2207362.

6. Huang Z, Chen Y, Peng J *et al.* *J. Mater. Chem. A.* 2023; **11**(13): 7201-7212.

7. Ji B, Zhou Q, Hu B *et al.* *Adv. Mater.* 2021; **33**(27): 2100859.

8. Keum K, Kwak JY, Rim J *et al.* *Nano Energy*. 2024; **122**: 109342.

9. Han R, Liu Y, Mo Y *et al.* *Adv. Funct. Mater.* 2023; **33**(51): 2305531.

10. Zhang C, Yang Q, Meng X *et al.* *Adv. Sci.* 2023; **10**(33): 2303418.

11. Qiu J, Yu X, Wu X *et al.* *Small*. 2023; **19**(15): 2205324.

12. Chen M, Ouyang J, Jian A *et al.* *Nat. Commun.* 2022; **13**(1): 7097.

13. Keum K, Cho SS, Jo J-W *et al.* *Iscience*. 2022; **25**(4): 104032.

14. Li X, Liu Y, Ding Y *et al.* *ACS Appl. Mater. Interfaces.* 2024; **16**:12974-12985.

15. Berman A, Hsiao K, Root SE *et al.* *Sci. Adv.* 2024; **10**(40): eadq8866.

16. Zhang C, Wang Z, Zhu H *et al.* *Adv. Mater.* 2024; **36**(4): 2308520.

17. Basarir F, Haj YA, Zou F *et al.* *Adv. Funct. Mater.* 2024; **34**(39): 2403268.

18. Wu L, Li X, Choi J *et al.* *Adv. Funct. Mater.* 2024; **34**(26): 2312370.

19. Guo H, Tan YJ, Chen G *et al.* *Nat. Commun.* 2020; **11**(1): 5747.

20. Huang J, Tang X, Wang F *et al.* *Adv. Eng. Mater.* 2022; **24**(9): 2101767.

21. Boutry CM, Negre M, Jorda M *et al.* *Sci. Robot.* 2018; **3**(24): eaau6914.

22. Su Z, Xu D, Liu Y *et al.* *ACS Appl. Mater. Interfaces* 2023; **15**(26): 32002-32010.

23. Hu H, Wang D, Tian H *et al.* *Adv. Funct. Mater.* 2022; **32**(8): 2109076.

24. Patil D, Liu S, Ravichandran D *et al.* *Small*. 2024: 2402432.

25. Fan Q, Miao J, Liu X *et al.* *Nano Lett.* 2022; **22**(2): 740-750.
